# Maleimide functionalized polycaprolactone micelles for glutathione quenching and doxorubicin delivery†

**DOI:** 10.1039/d4sc01625d

**Published:** 2024-05-24

**Authors:** Godwin K. Babanyinah, Abhi Bhadran, Himanshu Polara, Hanghang Wang, Tejas Shah, Michael C. Biewer, Mihaela C. Stefan

**Affiliations:** a Department of Chemistry and Biochemistry, University of Texas at Dallas Richardson TX USA mihaela@utdallas.edu

## Abstract

High glutathione production is known to be one of the defense mechanisms by which many cancer cells survive elevated oxidative stress. By explicitly targeting glutathione in these cancer cells and diminishing its levels, oxidative stress can be intensified, ultimately triggering apoptosis or programmed cell death. Herein, we developed a novel approach by creating maleimide-functionalized polycaprolactone polymers, specifically using 2,3-diiodomaleimide functionality to reduce the level of glutathione in cancer cells. Polycaprolactone was chosen to conjugate the 2,3-diiodomaleimide functionality due to its biodegradable and biocompatible properties. The amphiphilic block copolymer was synthesized using PEG as a macroinitiator to make corresponding polymeric micelles. The resulting 2,3-diiodomaleimide-conjugated polycaprolactone micelles effectively quenched glutathione, even at low concentrations (0.01 mg mL^−1^). Furthermore, we loaded these micelles with the anticancer drug doxorubicin (DOX), which exhibited pH-dependent drug release. We obtained a loading capacity (LC) of 3.5% for the micelles, one of the highest LC reported among functional PCL-based micelles. Moreover, the enhanced LC doesn't affect their release profile. Cytotoxicity experiments demonstrated that empty and DOX-loaded micelles inhibited cancer cell growth, with the DOX-loaded micelles displaying the highest cytotoxicity. The ability of the polymer to quench intracellular GSH was also confirmed. This approach of attaching maleimide to polycaprolactone polymers shows promise in depleting elevated glutathione levels in cancer cells, potentially improving cancer treatment efficacy.

## Abstract 2

High glutathione production is known to be one of the defense mechanisms by which many cancer cells survive elevated oxidative stress.

## Graphical abstract

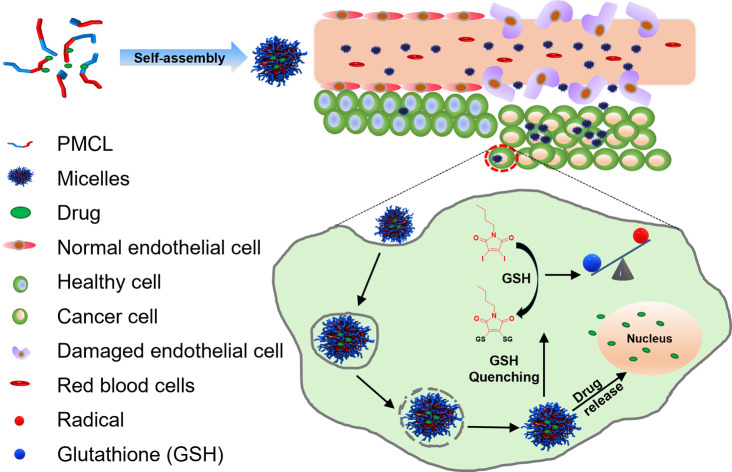

## Introduction

The synthesis of small organic molecules with anticancer activity is on the rise, as is the use of nanocarriers for drug delivery.^1–9^ Bridging these two fast-growing fields to design novel materials to accelerate cancer therapy is worth pursuing. Among many reported small organic molecules with anticancer properties, molecules that promote the build-up of inherent suicidal chemical species such as reactive oxygen species (ROS) within the microenvironment of the cancer cells are interesting yet under-reported.^10,11^ ROS are highly reactive oxygen-containing molecules such as superoxide anion (
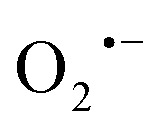
), hydrogen peroxide (H_2_O_2_), and hydroxyl radical (HO˙), that are often produced as a by-product from many cellular metabolic pathways. Higher levels of ROS are commonly evident in cells with fast cell proliferating rates, such as cancer cells.^12^ If the concentration levels of ROS remain unchecked, it results in oxidative stress, when the levels of ROS overwhelm the antioxidant defense system, ultimately leading to cellular damage. Such cellular damage arises when the ROS reacts with and damages macromolecules and cellular structures such as nucleic acids, proteins, and cell membranes, leading to cellular apoptosis.^13,14^ In response to the oxidative stress, cells produce several antioxidants such as glutathione (GSH) to neutralize the damaging effect of the ROS.^15–17^

GSH is a tripeptide consisting of cystine and glycine, widely known to be the most abundant antioxidant produced in the body. Therefore, GSH is considered the main defense mechanism against oxidative stress exerted by ROS.^18,19^ Since the production of GSH is linked with the levels of ROS, higher concentrations of ROS trigger elevated production of GSH. The scavenging activity of GSH involves the conversion of GSH into its oxidized form, glutathione disulfide (GSSG). Subsequently, GSH is regenerated from the GSSG to restart the redox cycle.^12,15,19,20^ Since high amounts of ROS are produced in cancer cells, higher concentrations of GSH are evident in cancer cells. For instance, the concentration of GSH can be as high as 10 mM, notably higher than observed in normal healthy cells (2 to 20 μM).^21^ Moreover, It is thought that the GSH concentration can go higher in drug-resistant cancer cells.^22–24^ This implies that cancer cells not only produce elevated amounts of GSH to fight oxidative stress but also to resist chemotherapy agents. Since the reduction of GSH level elevates oxidative stress and can potentially fight against drug resistance developed by cancer cells, specifically targeting GSH in cancer cells is one of the main strategies in contemporary anticancer drug discoveries.

Chemical agents such as iodoacetate, l-buthionine sulfoximine (BSO), sulforaphane, and diethylmaleate are reported to possess a GSH-depleting effect, hence their presence in cancer cells amplifying the intracellular oxidative stress thereby accelerating cancer cell death.^15,24,25^ These agents render the GSH non-reductive – inability to neutralize ROS – or reduce the biosynthesis of GSH causing accumulation of ROS in the cancer cell.^26–30^ Many GSH-quenching agents, such as aforementioned, are often non-specific and non-selective and thus can consume GSH in both cancer and healthy cells, potentially leading to undesirable side effects.^15,31,32^ Improving the selectivity of GSH-depleting agents to target only cancer cells is challenging. Such a challenge is not unique to GSH-depleting agents but many commercially available anticancer drugs, such as doxorubicin, whose numerous side effects are attributed to its non-selectivity.^33^ Besides, most GSH-depleting agents are hydrophobic and could potentially face challenges such as biodistribution and bioavailability, limiting their biological applications. One logical approach is attaching targeting agents to improve their selectivity and attaching water-soluble functional groups to improve their general water-solubility without compromising their GSH-depleting capabilities.^11^ The most common approach to improving both the specificity and selectivity and water-solubility is the use of nanocarriers as drug delivery systems.^34–36^ For instance, water-soluble nanocarriers within the sizes of 20–200 nm are known to have enhanced permeability and retention (EPR) effect due to the defective angiogenic vasculature and the poor lymphatic drainage system in solid tumors. Therefore, a drug-loading system loaded with therapeutic agents of this size can selectively enter and accumulate in tumors relative to healthy cells, thereby minimizing their lethal effect on the healthy cell while exerting full toxicity toward the cancer cell.^37–40^ For instance, Zhou *et al.* explored this strategy by loading maleimides, a GSH quenching agent, into liposomes to promote cancer cell death.^41^

Several nanocarriers, such as liposomes, dendrimers, hydrogels, and polymeric nanoparticles/micelles, are frequently investigated for drug delivery.^42–44^ While these nanocarriers have advantages and limitations, polymeric micelles display outstanding potential for drug delivery applications due to their tunable properties, ease of preparation, high solubilization characteristics, and excellent biocompatibility.^45–49^ These micelles are usually formed as the amphiphilic block copolymers self-assemble in aqueous solution. Aliphatic polyesters such as poly(ε-caprolactone) (PCL), poly(glycolide) (PGA), poly(lactide) (PLA), and poly(lactide-*co*-glycolide) (PLGA) are predominantly used to make polymeric micelles due to their biocompatibility and biodegradability.^8,50,51^ Among these, PCL is reported to be more desirable due to its good mechanical properties, high drug permeability, and slower rate of degradation.^8,52^ Additionally, the properties of PCL can easily be tuned through substitution at either the α- or γ-position of the ε-caprolactone (CL) monomer.^53–57^ For instance, functional groups such as cancer targeting agents, fluorescent agents, cancer therapeutic agents, and other groups that increase the drug-loading capacity of micelles can easily be substituted.^4–6,58–64^

This report is deemed to bridge the gap between the synthesis of small organic anticancer agents and the use of polymeric micelles for anticancer drug delivery. Since maleimides are reported to react with free thiol, their ability to deplete intracellular GSH is under-reported. Zhou *et al.*^41^ demonstrated that maleimide can deplete GSH by reacting with the free thiol, which is the active site for ROS inhibition.^41,65,66^ This GSH quenching occurred through an irreversible Michael addition reaction between the free thiol and the maleimide. However, the use of such maleimide for GSH depletion is not efficient since only one GSH can be consumed per maleimide (1 : 1 equivalent). Therefore, to achieve higher cancer cell death, a large amount of maleimide would have to be loaded, which is also limited by the loading capacity of the liposome. Designing novel polymeric micelles whose hydrophobic block is conjugated with maleimides capable of reacting with more than one GSH at a time can address this concern since such an approach lessens the problem of loading capacity while improving the system's efficiency. Additionally, this system will free up the micelle core for further use in loading other hydrophobic anticancer drugs, which can effectively amplify the overall therapeutic effect of chemotherapy.

**Fig. 1:**
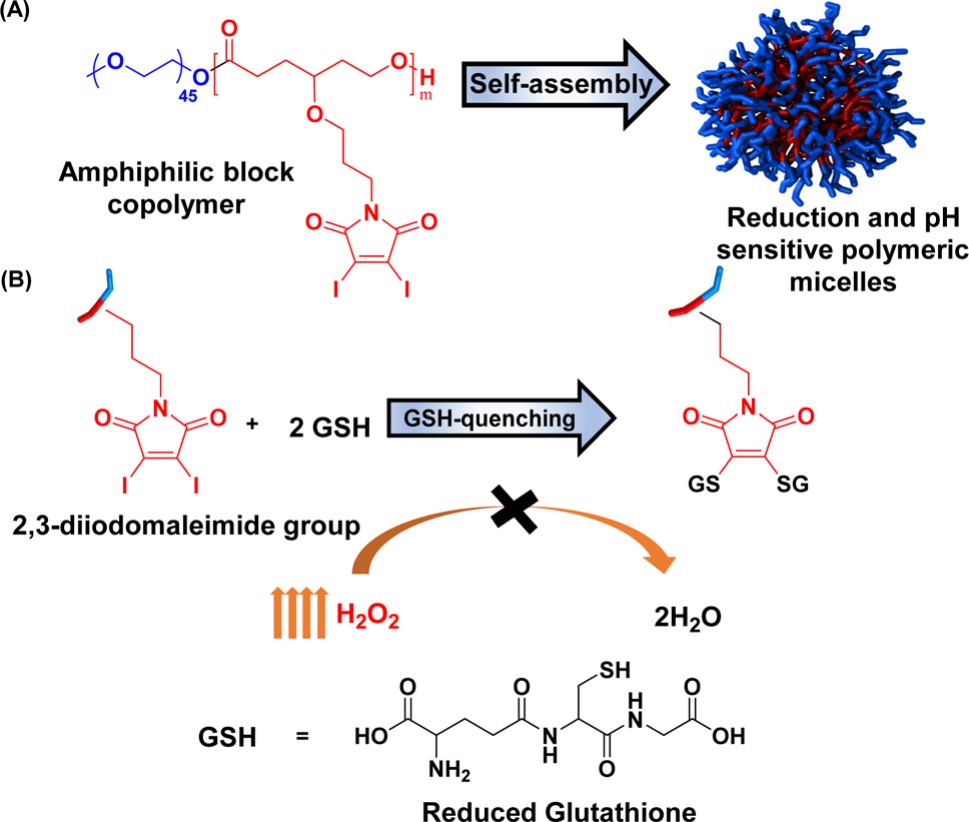
Schematic representation of (a) preparation of micelles and (b) their reactivity towards glutathione.

Herein, we report the maleimide-functionalized PCL synthesized through the ring-opening polymerization of their corresponding 2,3-diidomaleimide γ-substituted CL monomer. The 2,3-diiodomalemide functionality was chosen explicitly over the unsubstituted maleimides due to their rapid reactivity toward thiols^67^ and their ability to quench two GSH at a time (Fig. 1) instead of one in the case of the unsubstituted maleimides.^41,68,69^ Furthermore, to facilitate passive targeting through an EPR effect, polymeric micelles were designed from these polymers using PEG as the hydrophilic component. Polymeric micelles bearing a PEG shell can stealthily circulate in the bloodstream, extending their half-life in the body.^70,71^ The obtained micelles were further utilized to load the anticancer drug doxorubicin (DOX). The GSH quenching effect of the amphiphilic polymer was tested. Both DOX-loaded and empty micelles were also characterized. The drug loading capacity and release profile were determined at both physiological pH and low pH – a typical pH of most solid tumors.^72–74^ To better define the properties of the obtained micelles, previously reported poly(ethylene glycol)-*b*-poly(γ-benzyl-ε-caprolactone) PEG-*b*-PBCL, represented as PBCL in this report, micelles are used for comparison.^75^

## Experimental

### Materials

Chemicals needed for this study were purchased from either Fisher Scientific or Sigma-Aldrich and were used without purification except for Sn(Oct)_2_, which was purified through vacuum distillation. The polymerization was carried out under a nitrogen atmosphere using toluene from a sodium/benzophenone ketyl distillation system. Schlenk flask and syringes used for the polymerization were kept at 120 °C for 24 h and cooled in a desiccator before use. The synthesis of the monomer and the polymerization was guided by previously published procedures with slight modifications.^5,6,76–78^

### Analysis

A Bruker AVANCE III™ (500 MHz) nuclear magnetic resonance (NMR) instrument was used to collect the NMR ^1^H and ^13^C spectra at 25 °C of the monomer, its intermediates, and the polymers dissolved in deuterated chloroform (CDCl_3_). The dispersity (*Đ*) and the number average molecular weights of the polymers were determined using Viscotek VE 3580 size exclusion chromatography (SEC) with Viscotek columns (T6000M) and refractive index (RI) detector. THF (HPLC grade) was used as the eluent with a flow rate of 1 mL min^−1^ at 30 °C with GPC max as the sample module, and RI detection calibration was based on polystyrene standards. The molar mass of the monomer was determined by matrix-assisted laser desorption ionization time-of-flight (MALDI-TOF) spectra using Shimadzu biotech axima confidence in reflectron_HiRes mode with dithranol as the matrix. A Malvern Zetasizer instrument with a 4 mW He–Ne laser (633 nm) and an avalanche photodiode (APD) detector was employed for dynamic light scattering (DLS) measurements. Measurements were obtained with a detector angle of 173° at 25 °C. Cytotoxicity, cellular uptake, and intracellular GSH imaging were determined with BioTek Cytation 3 cell imaging multi-mode reader. This reader was also used for all the UV-vis analyses. Tecnai G2 Spirit Biotwin microscope operating at 120 keV was employed to record TEM images, and images were analyzed using Gatan Digital Micrograph software. TEM samples were prepared by treating the copper mesh grid with 1 mg mL^−1^ aqueous polymer micelle solution for 2 min, then staining with 2% Phosphotungstic acid for 30 s.

### Synthesis of homopolymer

In an oven-dried 10 mL Schlenk flask, dried monomer (200 mg, 0.385 mmol), benzyl alcohol (0.83 mg, 0.008 mmol), and Sn(Oct)_2_ (3.12 mg, 0.008 mmol) in dry toluene (0.2 mL) were added inside a nitrogen-filled glove box according to our previous reports.^3,4,76^ The flask was tightly sealed and then placed in a preheated thermostat-controlled oil bath at 110 °C. The consumption of monomer was monitored using ^1^H NMR spectroscopy and the monomer was consumed fully after 6 h. The polymerization mixture was then brought to room temperature and precipitated in cold methanol. The polymer was then dissolved in a minimum amount of THF and precipitated again in cold methanol. The purification process was repeated three times and then dried under vacuum to yield the homopolymer as a yellow viscous liquid (yield, 180 mg). ^1^H NMR (500 MHz, CDCl_3_) *δ* 4.14 (t, 2*H*), 3.73 (t, 2*H*), 3.49–3.38 (m, 3*H*), 2.36 (t, 2*H*), 1.89–1.72 (m, 6*H*). ^13^C NMR (500 MHz, CDCl_3_) *δ* 173.55, 166.54, 128.68, 128.40, 117.60, 75.75, 66.54, 61.37, 38.04, 32.96, 29.83, 29.07, 28.90.

### Synthesis of amphiphilic block copolymer (PMCL and PBCL)

To an oven-dried 10 mL Schlenk flask, dried monomer (1.0 g, 2 mmol), PEG_2000_ (0.128 g, 0.06 mmol), and Sn(Oct)_2_ (0.026 g, 0.06 mmol) in dry toluene (0.5 mL) were added inside a nitrogen-filled glove box based on our previous reports.^3,4,76^ The flask was tightly sealed and then placed in a preheated thermostat-controlled oil bath at 110 °C. The consumption of monomer was monitored using ^1^H NMR spectroscopy and the monomer was consumed fully after 6. The polymerization mixture was then brought to room temperature and precipitated in cold methanol. The polymer was then dissolved in a minimum amount of THF and precipitated in cold methanol. The same procedure reported previously was followed to synthesize PBCL.^4^ The process was repeated three times and dried under vacuum to yield PMCL as a yellow viscous liquid (yield, 0.9 g). ^1^H NMR (500 MHz, CDCl_3_) *δ* 4.13 (t, 2*H*), 3.72 (t, 2*H*), 3.63 (s, 4*H*), 3.42 (tdd, 3*H*), 2.36 (q, 2*H*), 1.90–1.67 (m, 6*H*). ^13^C NMR (126 MHz, CDCl_3_) *δ* 173.59, 166.56, 117.60, 75.79, 70.72, 70.68, 66.57, 61.40, 38.06, 32.98, 29.85, 29.83, 29.08, 28.92.

### Spectra analysis of PMCL-GSH reaction

The absorbance peak shift when PMCL reacts with GSH was observed and determined through UV-vis spectroscopy. In this setup, 200 μL (0.1 mg mL^−1^) PMCL was added to 100 μL (10 mM) GSH solution. In another vial, 200 μL (10 mM) of Ellman's reagent 5,5′-dithiobis-(2-nitrobenzoic) acid (DTNB) was added to 100 μL GSH solution. The solutions were allowed to incubate for about 30 minutes at room temperature. The absorbance of 200 μL of each solution, PMCL, PMCL-GSH, Ellman-GSH, and GSH was read from 300 nm to 700 nm.

### Solution-phase GSH quenching test

The ability of the PMCL to quench GSH was tested using Ellman's assay. In this process, 100 μL GSH (10 mM) prepared in PBS (1x, pH 7.4) was added to different concentrations of the polymer solution and incubated for 30 minutes. Afterward, 300 μL (10 mM) of Ellman's reagent was added to the solution to react with any remaining GSH. The absorption peak at 412 nm was used to quantitatively determine the amount of the GSH quenched. This calculation was based on a GSH calibration curve (Fig. S18†) obtained using Ellman's reagent.^79–81^

## Preparation of empty and loaded micelle

Empty micelles were prepared by solvent evaporation method.^3,4,6,61,76,82^ Polymer weighing 5.0 mg was dissolved in 1.5 mL THF and was added dropwise to 5.0 mL deionized (DI) water while homogenizing for 30 min. The polymer solution was then kept on a vortex with gentle agitation to slowly remove the organic solvent to obtain a final polymer concentration of 1 mg mL^−1^. The DOX-loaded micelles were also prepared following this method with slide modification.^3,76^ To prepare the DOX solution, 1.0 mg of DOX HCl was dissolved in 1.0 mL of THF, and 3.0 equivalents of triethylamine were added to neutralize the DOX HCl. A solution of polymer to DOX of a 10 : 1 weight ratio was prepared. After mixing thoroughly, the solution was added dropwise to 5.0 mL DI water while homogenizing. After 30 minutes of homogenization, the remaining THF was slowly evaporated using a vortex under slow agitation. The micelle solution was then transferred into a SnakeSkin® dialysis bag with a size cut-off point of 3500 Da and dialyzed in 500 mL PBS (pH 7.4) for about 24 h. The dialysis media was changed every 8 h to remove traces of THF and DOX that were not encapsulated. The DOX-loaded micelles were then filtered with a 0.22 μm Nylon syringe filter to obtain the final DOX-loaded micelles. The drug loading capacity (DLC) and efficiency (DLE) of the micelles were then calculated by the equation below. The size and morphology of both the DOX-loaded and empty micelles as well as their time-dependent stability were determined.
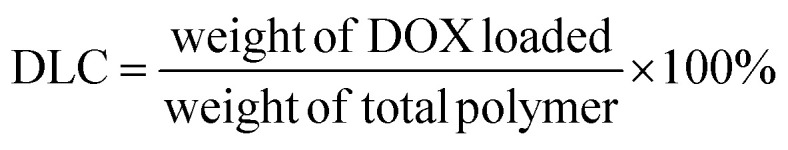

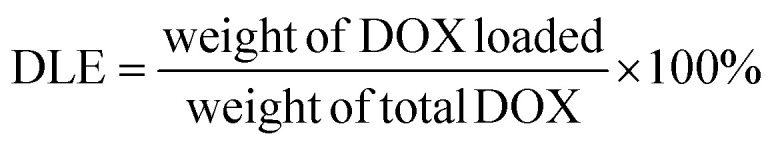


### Critical micelle concentration determination

The critical micelle concentration (CMC) of the polymer was determined through fluorescence microscopy by using pyrene as the hydrophobic fluorescent probe. In this study, serial concentrations of the polymer were prepared in THF, and 25 μL of pyrene stock solution (1.24 mmol mL^−1^) was added. The polymer/pyrene solution was added to 10 mL DI water in drops to make a serial polymer concentration from 1.0 mg mL^−1^ to 1 × 10^−6^ mg mL^−1^ while the solution was under a slow vortex. The final solution was allowed to vortex to evaporate any residual THF. The excitation spectra of the pyrene from 300 to 360 nm were taken at a fixed emission of 390 nm for each solution using a fluorescence spectrometer. The CMC was graphically determined by plotting the intensity ratio at 337.5 nm to 334.5 nm as (*I*_337.5_/*I*_334.5_) against the log of the polymer concentration.

### Size and morphology

The micelle size was determined using a DLS instrument, Malvern Zetasizer Nano ZS. The micelles were prepared as described above with a concentration of 1 mg mL^−1^ which is above the CMC. The micelle solution was equilibrated at 25 °C and the hydrodynamic diameters (*D*_h_) of the micelles were determined. The morphology of the micelles was determined using TEM, 2 wt% phosphotungstic acid (PTA) stain, and copper mesh grid. To achieve this, 10 μL of the micelle solution and 10 μL of PTA were placed on a separate parafilm. A glow-discharged copper mesh grid was carefully laid horizontally on the sample for 2 minutes, followed by exposure to the stain for 1 minute. After each step, the excess solution was removed from the grid by gently dabbing the edge with filter paper. The grid was then placed on a Petri dish with the dark side facing up overnight and subsequently analyzed using TEM.

### Drug release study

The *in vitro* pH-dependent DOX release from the polymeric micelles was determined at physiological conditions (pH of 7.4, 37 °C) and tumor-mimicking environment (pH 5.0, 37 °C) using PBS. In the set-up, 4.0 mL of the DOX-loaded micelles was transferred into a 3500 Da dialysis bag and incubated in 10 mL release media (PBS). This was done for both pH conditions. For the effect of the GSH on the release of DOX, the release media was supplemented with GSH (10 mM). At certain time intervals, 0.3 mL of the release media was drawn, and 0.3 mL of fresh media was replaced. The amount of the DOX released was determined through UV-vis analysis. The cumulative DOX release was then calculated from the equation below, where *m*_DOX_ is the amount of DOX released into the media, *V*_0_ is the volume of the release media (*V*_0_ = 10.0 mL), *V*_e_ is the volume of the replaced media (*V*_e_ = 0.3 mL), and *C*_*n*_ is the concentration of DOX in the sample.
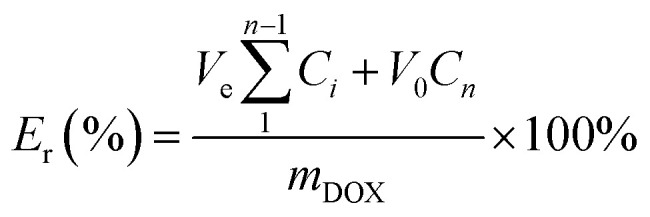


### Cell culture

MDA-MB-231 cells (ATCC® HTB-26™) were obtained from American Type Culture Collection (ATCC). Cell lines were cultured under standard conditions (37 °C and 5% CO_2_) in Dulbecco's Modified Eagle Medium (DMEM, Sigma-Aldrich) supplemented with 10% fetal bovine serum (FBS, Gibco™, Fisher Scientific) and 1% penicillin/streptomycin (Gibco™, 10 000 U mL^−1^, Sigma-Aldrich) and split using trypsin–EDTA upon confluence. Cells were maintained at 37 °C and 5% CO_2_ in a humidified incubator and were routinely sub-cultured at *ca.* 80% confluency. Trypsin–EDTA, 4′,6-diamidino-2-phenylindole (DAPI) and 3-(4,5-dimethylthiazol-2-yl)-2,5-diphenyltetrazolium bromide (MTT) were purchased from either Fisher Scientific or Sigma-Aldrich.

### Cell viability assay

MDA-MB-231 Breast cancer (ATCC, HTB-26) cells, Dulbecco's Modified Eagle Medium (DMEM) supplemented with 10% FBS and 1.0% penicillin/streptomycin (pen/strep) was used as the culture media and 3-(4,5-dimethylthiazol-2-yl)-2,5-diphenyltetrazolium bromide (MTT) as the staining agent. The cells were seeded in a 96-well plate with each plate having 1 × 10^4^ cells. The cells were allowed to adhere to the bottom of the wells by incubation at 37 °C, 5% CO_2_ for 24 h. The media was replaced with fresh media and then treated with 40 μL of different concentrations (0.5 mg mL^−1^ to 0.03 mg mL^−1^) of the empty and drug-loaded micelles. The control wells were treated with 40 μL pre-warmed PBS. The cell was then incubated for another 24 h in the same conditions. The cells were then rinsed with 100 μL PBS. Afterward, 30 μL (5 mg mL^−1^) MTT solution was added and further incubated for another 4 h. After the media was removed 170 μL DMSO was added to dissolve the formazan crystals. The absorption was recorded at 540 nm and normalized to the intensity of the control cells (*N* = 5, ± standard deviation). The statistical analysis was performed in Excel by using one-way ANOVA and Student's *t*-test. The *p*-values of less than or equal to 0.05 were statistically significant.

### Cellular uptake

MDA-MB-231 cells were grown in a 35 mm 24-well plate at a cell density of 250 000 cells per well and incubated for 24 h at 37 °C, 5% CO_2_. The media was replaced, and 40 μL DOX-loaded micelles (0.25 mg mL^−1^) were added to the cells. The cells were further incubated in the same condition for 4 h. Afterwards, the media was removed, and the cells were washed three times with pre-warm PBS and were then fixed with 100 μL 4% paraformaldehyde. After incubating for 10 minutes, the cells were washed three times with PBS, and the nucleus was counter-stained with 4′,6-diamidino-2-phenylindole (DAPI, blue) according to the manufacturer's recommended protocol. The images were taken using the BioTek Cytation 3 fluorescent microscope to determine the cellular uptake of the micelles.

### Intracellular GSH quenching

The detection of intracellular GSH levels in the cancer cell lines was determined using a slightly modified procedure from a previously reported method. After seeding the 96 well plates with MDA-MB-231 cells and incubating at 37 °C, 5% CO_2_ for 24 h, the media was replaced, and the cells were treated with 40 μL of different concentrations (0.5 mg mL^−1^ to 0.03 mg mL^−1^) of both empty and DOX-loaded PMCL micelles while the control cells were treated with Dulbecco's phosphate-buffered saline (DPBS) without Ca^2+^ or Mg^2+^. The cells were further incubated for 24 h. The media was replaced with 100 μL of the ThiolTracker Violet dye (20 μM) and further incubated for 30 minutes. Afterward, the cells were washed three times with DPBS buffer, and the level of the intracellular GSH was determined by measuring the emission intensity of the ThiolTracker Violet at 515 nm at an excitation wavelength of 415 nm. The intensity was normalized against the intensity of the untreated cells (*N* = 5, ± standard deviation). The statistical analysis was performed in Excel by using one-way ANOVA and Student's *t*-test. The *p*-values of less than or equal to 0.05 were statistically significant. The level of GSH present after the treatment was also imaged using BioTek Cytation 3 fluorescent microscope.

### Statistical analysis

All the data are expressed as a mean ± standard deviation as presented by the bars in the graphs. The statistical analysis depicting the significance of the differences between exposures and the controls was performed using both Student's *t*-test and one-way ANOVA on MS Excel where a *p*-value < 0.05 was regarded as statistically significant.

## Results and discussion

### Synthesis of monomer

**Scheme 1:**
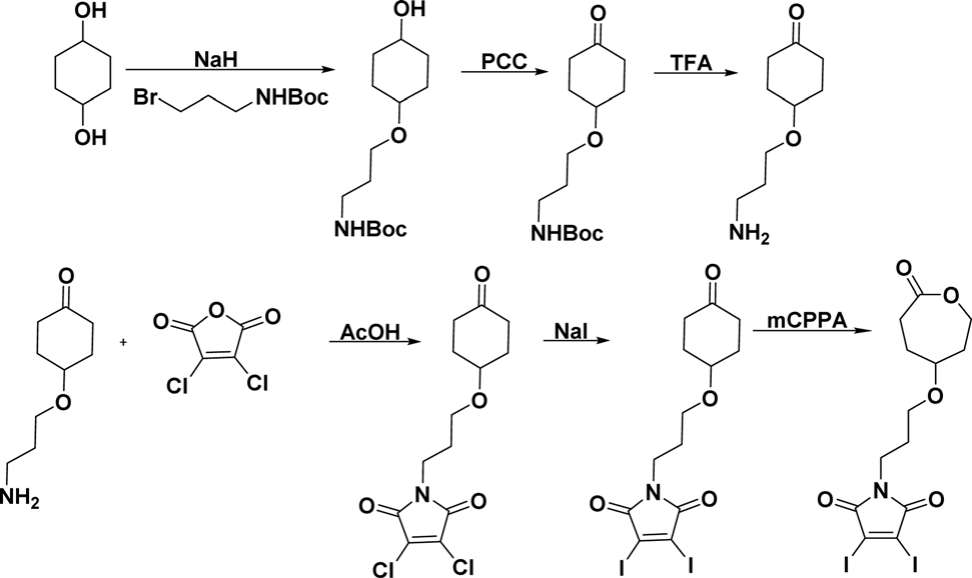
Reaction scheme for the synthesis of DIMCL monomer.

The novel monomer was designed by functionalizing ε-CL at the γ-position with a GSH-quenching group. Diiodo maleimide moiety was chosen as the GSH-quenching group since it can react with two equivalents of thiol groups. The diiodomaleimide moiety was attached to the γ-position of ε-CL monomer through a propyloxy linker. The synthetic steps leading to the monomer are presented in Scheme 1 and were executed through a slight modification of previously reported methods.^3,4,76^ The starting material, 1,4-cyclohexanediol, was first treated with one equivalent of sodium hydride (NaH), followed by a mono-substitution reaction with 3-(boc-amino)propyl bromide. The resulting 3-(boc-amino)propyl-substituted cyclohexanol was subsequently oxidized using pyridinium chlorochromate (PCC) to yield the corresponding cyclohexanone. The Boc protecting group was then removed with excess trifluoroacetic acid. The resulting 3-amino propyl-substituted cyclohexanone was then reacted with 2,3-dichloro maleic anhydride, forming 2,3-dichloro maleimide propyl-substituted cyclohexanone (Scheme S3†). This was followed by a substitution reaction with sodium iodide (NaI), replacing chlorine atoms with iodine, resulting in 2,3-diiodo maleimide propyl-substituted cyclohexanone (Scheme S4†). The replacement of chlorine with iodine was due to the higher reactivity of 2,3-diiodomaleimide toward thiols and a slower rate of *N*-propylldiiodomaleimide hydrolysis.^67^ Finally, Baeyer-Villiger oxidation of the 2,3-diiodomaleimide propyl-substituted cyclohexanone with *m*-chloroperoxybenzoic acid (*m*CPBA) led to the formation of the desired 2,3-diiodo maleimide propyl substituted CL (DIMCL) monomer. All the intermediates were characterized using NMR (^1^H and ^13^C) spectroscopy (Fig. S1–S10†). The molecular weight of the DIMCL monomer was confirmed with MALDI-TOF mass spectrometry (Fig. S11†).

### Synthesis of polymer

**Scheme 2:**
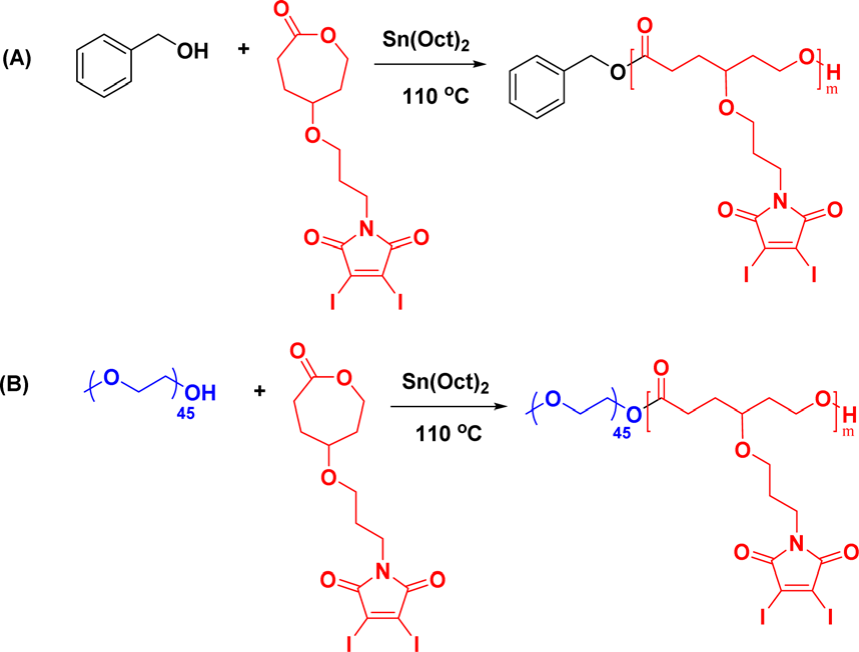
Synthesis of (A) homopolymers and (B) amphiphilic block copolymers of DIMCL through ROP.

**Fig. 2:**
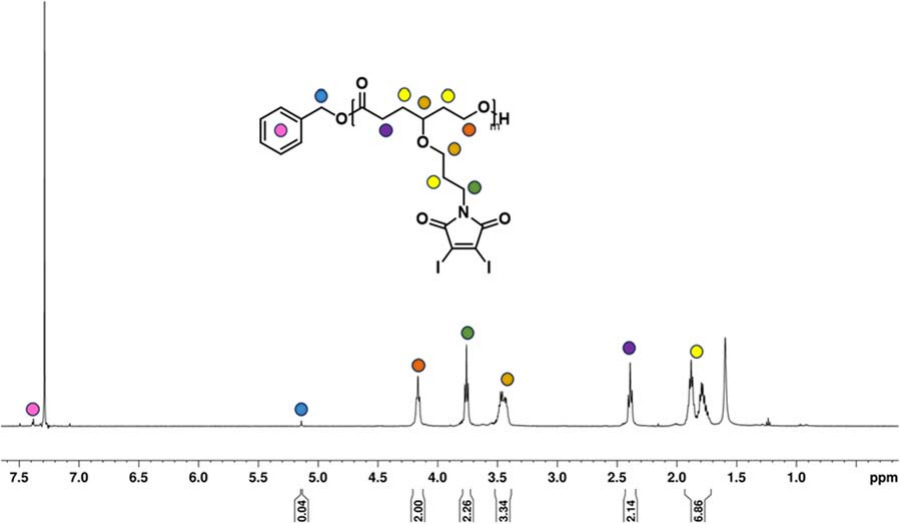
^1^H NMR spectrum of homopolymer synthesized from DIMCL monomer.

Given the novelty of the DIMCL monomer, it was imperative to investigate whether the monomer is polymerizable in a coordination-insertion ring-opening (ROP) polymerization fashion. This was accomplished through the ROP of the DIMCL monomer using Sn(Oct)_2_ as the catalyst and benzyl alcohol as the initiator (Scheme 2a). The reaction was performed under a nitrogen atmosphere and a complete monomer consumption was observed after 6 h. The pure polymer was characterized using NMR – ^1^H NMR (Fig. 2) and ^13^C NMR (Fig. S14†) and SEC (Fig. S16A†). A 50 : 1 : 1 mole ratio (monomer: catalyst: initiator) was used to produce the polymer with a desired degree of polymerization (DP_n_) of 50 and a molecular weight of 26 000 g mol^−1^. ^1^H NMR shows that the obtained polymer has a similar molecular weight of 25 000 g mol^−1^ and a DP_n_ of 48. The molecular weight of the polymer was also confirmed through ^1^H NMR analysis by multiplying the DP_n_ of polymers from the ^1^H NMR, by the molecular weight of monomers and then adding the molecular weight of the initiator. The DP_n_ of the polymer was determined by the ratio of the integrations of the methylene protons of the propyloxy linker (∼4.15 ppm) and the methylene protons of the benzyl end group (∼5.1 ppm). The SEC analysis gave an unimodal polymer distribution with a dispersity of 1.25 and an average molecular weight of 9600 g mol^−1^. The molecular weights determined by SEC analysis appear lower than those estimated through ^1^H NMR. This discrepancy could potentially be attributed to the variations in the hydrodynamic volume of the polymer and the polystyrene reference calibration standard used.^4,6,83^

**Fig. 3:**
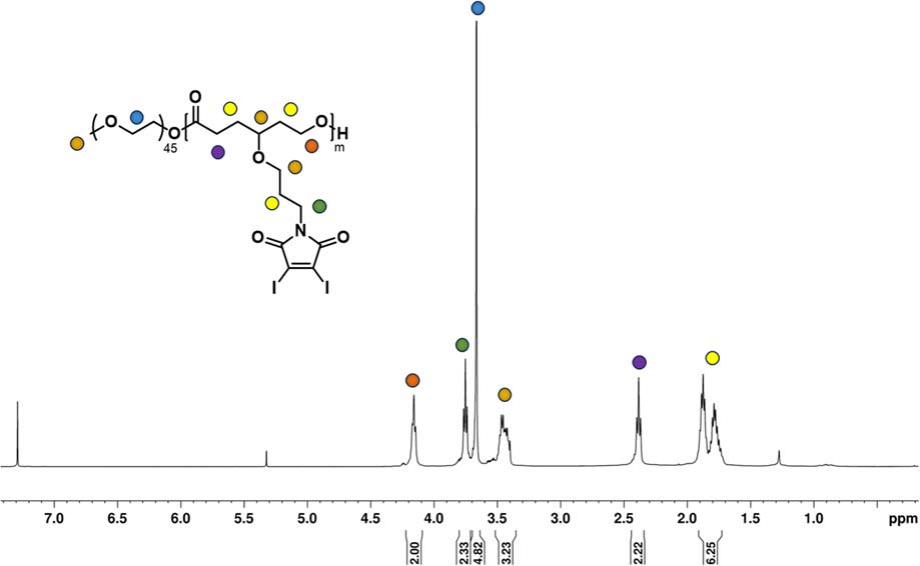
^1^H NMR spectrum of amphiphilic block copolymer PMCL.

Once the homopolymerization of the DIMCL monomer was successfully confirmed and characterized, we employed the same ROP conditions to synthesize amphiphilic block copolymers using PEG_2000_ as a macroinitiator (Scheme 2b). The monomer was consumed after stirring for 6 h at the conditions mentioned above. The complete polymerization was also confirmed *via*^1^H NMR spectroscopy. The purification of the obtained amphiphilic block copolymer was done by precipitation method. The polymer was dissolved in THF and precipitated in chilled methanol. This process was repeated three times to remove any impurities. The purified final polymer (PMCL) was analyzed using ^1^H NMR (Fig. 3), ^13^C NMR (Fig. S15†), and SEC (Fig. S16b†). A 40 : 1 : 1 mole ratio (monomer: catalyst: initiator) was used to produce PMCL polymer with a desired DP_n_ of 40 and a molecular weight of 22 800 g mol^−1^. Since the DIMCL monomer has a relatively higher molecular weight and is more hydrophobic than similar hydrophobic CL monomers, the DPn of the PMCL polymer was deliberately maintained at a lower level compared to the homopolymers to ensure a balance between hydrophilic and hydrophobic blocks. The ^1^H NMR data revealed that the PMCL polymer has a molecular weight of 21 200 g mol^−1^ with a DP_n_ of 37. The molecular weight estimated from ^1^H NMR was calculated using the same method as the homopolymers. The DP_n_ of the polymer was determined by measuring the ratio of the integrations of the methylene protons of the propyloxy linker (∼4.15 ppm) and the ethylene protons (∼3.65 ppm) of the PEG_2000_ (∼45 DP_n_). SEC analysis gave unimodal polymer distribution with a dispersity of 1.37 and a number average molecular weight of 8500 g mol^−1^.

The PMCL amphiphilic block copolymers are synthesized as it is crucial for the preparation of the polymeric micelles. It is through this approach that we can effectively solubilize our polymer in aqueous conditions, enabling its application for GSH quenching and facilitating the loading of any hydrophobic anticancer drug molecule. For the comparative study regarding the ability of the PMCL micelles to form micelles, load, and deliver hydrophobic anticancer drugs into cancer cells, we also synthesized amphiphilic block copolymer P PBCL using PEG_2000_ macroinitiator which our group has previously reported.^75^

### Reactivity of monomer and PMCL towards glutathione

The ability of the 2,3-diiodomaleimide groups of the PMCL to react and quench thiol-containing molecules such as GSH was demonstrated by reacting the monomer with benzyl thiol as a GSH model. Since the monomer is hydrophobic and will not react with the hydrophilic GSH, the benzyl thiol soluble in organic solvent was used to demonstrate the quenching ability of the 2,3-diiodomaleimide group using NMR. Unlike maleimides, which undergo Michael addition reaction with thiol compounds to produce a single substituted product,^84–86^ each 2,3 diiodomaleimide group of the monomer reacted with two thiols and was confirmed by the ^1^H NMR (Fig. S12†) and ^13^C NMR (Fig. S13†). Interestingly, this reaction was rapid; the reaction was completed after 30 minutes, and the disubstituted product, which was yellow, constituted about 85% yield. This observation is consistent with numerous other reports that dihalomaleimides undergo halogen exchange reactions with free thiols to produce dithiomaleimides, with diiodomaleimide showing the fastest reaction rate.^87–89^

**Fig. 4:**
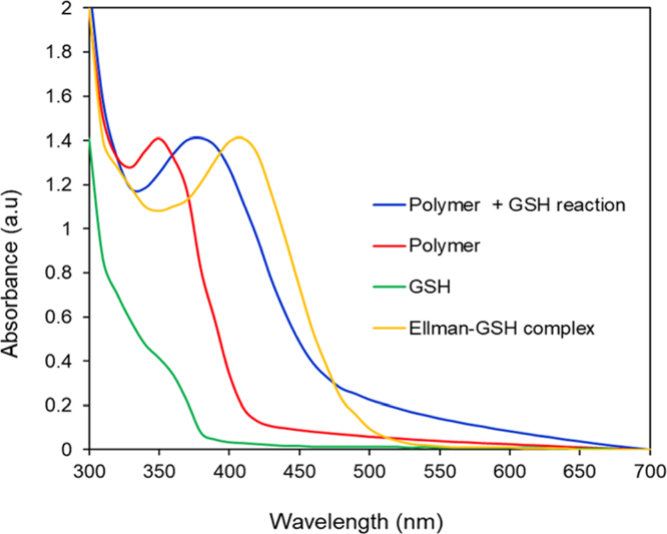
UV-vis analysis of the reactivity of GSH with PMCL polymer and Ellman's reagent. PMCL peak shifts from 350 nm to 380 nm upon reaction with GSH.

The PMCL is an amphiphilic polymer; hence, it self-assembles to form micelles in the presence of aqueous media where the 2,3-diiodomaleimide groups are buried in the core of the micelle. This was intentionally designed to protect the 2,3-diiodomaleimide groups from reacting with other molecules during the circulation of the micelle in the blood. However, some of the GSH can diffuse into the micelle core to react with the 2,3-diiodomaleimide groups. This is possible because GSH is reported to diffuse into hydrophobic environments.^90–92^ The reactivity of PMCL polymer towards GSH was observed using UV-vis spectroscopy. When the PMCL polymer was mixed with GSH in PBS and formed micelles, the two iodine atoms in the 2,3-diiodomaleimide groups of the polymer were replaced by the thiol group of GSH molecules as confirmed by the model reaction described above to produce di-glutathione maleimides. This change becomes prominently evident as the solution underwent a transition from a colorless to a yellow-colored solution after mixing for about 30 minutes. The PMCL polymer in DI water had a sharp absorbance peak at 350 nm coming from the 2,3-diiodomaleimide group. After the reaction with glutathione, this peak shifted to 380 nm confirming the reaction of the maleimide group with the GSH (Fig. 4).

### Solution-phase GSH-quenching effect by PMCL

**Fig. 5:**
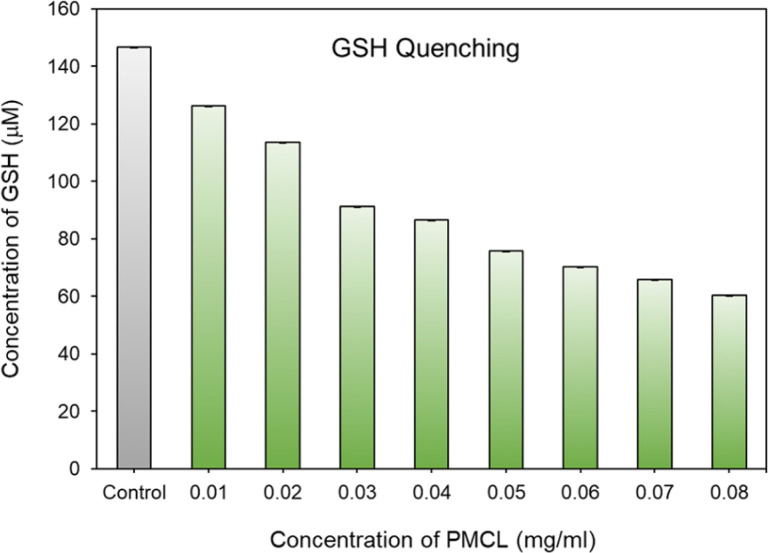
GSH quenching effect of PMCL.

To quantitatively analyze the amount of GSH quenched by PMCL, Ellman's assay was conducted. Ellman's assay is a characteristic test used to qualitatively or quantitatively determine the amount of free thiols present in a solution.^93,94^ By adding Ellman's reagent to a pretreated solution of PMCL/GSH, we quantified the amount of unreacted GSH present in the solution. This is possible because the unreacted GSH reacts with Ellman's reagent to produce a yellow 2-nitro-5-thiobenzoic acid which has its maximum absorbance peak at 412 nm. As reported, DTNB reacts with thiol in 1 to 1 molar ratio to produce 1 equivalent of 2-nitro-5-thiobenzoic acid. Hence the absorbance of the yellow 5-thionitrobenzoic acid is directly related to the amount of unquenched GSH present in the solution.^79–81^ Towards that, the ability of the polymer to quench GSH in a polymer concentration-dependent was determined by reacting the same amount of GSH with different polymer concentrations in a physiological condition. It was observed that the amount of unreacted GSH in the media kept decreasing as the concentration of the polymer (source of maleimide) increased (Fig. 5). The amount of the unquenched GSH was calculated using a pre-established GSH calibration curve (Fig. S18†). The ability of each repeating unit to react with more than one GSH molecule at a time makes this polymer more effective at depleting GSH in cancer cells than other approaches reported.^41^ From Fig. 5, a PMCL polymer concentration of 0.08 mg mL^−1^ shows that about 50% of the added GSH was quenched by the polymer. The high quenching effect at these polymer concentrations is because of the ability of each 2,3-diiodomaleimide repeat unit in the polymer backbone to quench two GSH at a time. This approach in GSH-mediated chemotherapy is desirable because such conjugation of the GSH-quenching functional group directly to the polymer hydrophobic block can increase the efficiency of its use to improve chemotherapy through its application as anticancer drug delivery. Moreover, the incorporation of the GSH-quenching group at the hydrophobic block, constituting the core of the micelle, ensures that the GSH-quenching group is shielded from other thiol-containing molecules from reaction with the maleimide during the circulation and biodistribution of the micelle. This way, the micelles reach the tumor site with the GSH-quenching group intact where its effect is expected to start.

### Critical micelle concentration determination

**Fig. 6:**
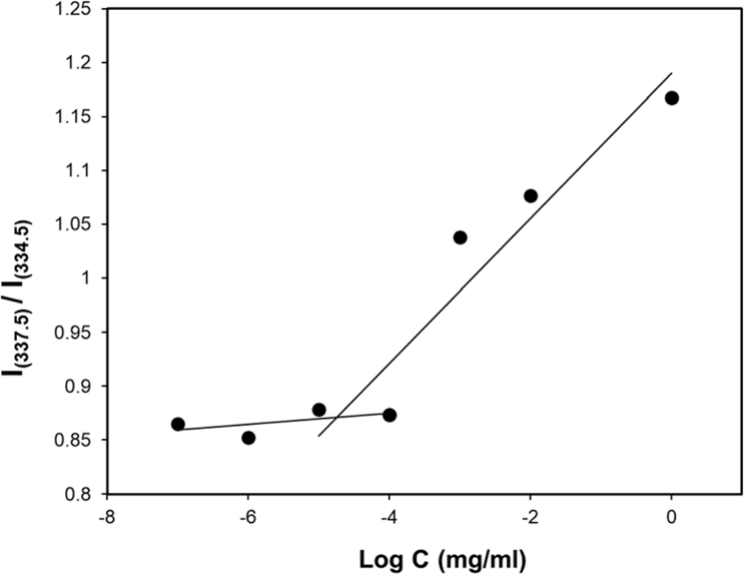
CMC determination of PMCL using the pyrene method where the ratio of fluorescent intensity of pyrene is plotted against polymer concentrations. The CMC of PMCL was determined to be 1 × 10^−5^ mg mL^−1^.

The concentration above which amphiphilic polymers self-assemble to form micelles in aqueous solution is called critical micelle concentration (CMC). This is an important parameter to measure micelle stability in both *in vitro* and *in vivo* since polymeric micelles undergo extensive dilution once introduced into the blood.^95^ It is imperative that micelles remain stable without disintegrating during biodistribution and circulation. In an event where the drug-loaded micelles fall below their CMC, the micelles will prematurely release the loaded drug before reaching the targeted site hence, undesirably exposing the drug to healthy cells. Therefore, amphiphilic polymers having lower CMC are more desirable. The CMC of the PMCL polymer was determined using fluorescence spectroscopy with pyrene as the fluorescent probe molecule. A series of polymer solutions with a constant pyrene concentration but varying polymer concentrations were prepared in PBS. As the environment of pyrene shifts from hydrophilic to hydrophobic, there is an observable transition in the excitation peak of pyrene fluorescence, shifting from 337.5 nm to 334.5 nm.^96^ The CMC was established by detecting this abrupt change in the fluorescence intensity ratio. The fluorescence intensity ratio was plotted against the concentration of the polymer as shown in Fig. 6. The CMC for the PMCL was found to be 1.10 × 10^−5^ mg mL^−1^ which is lower than PBCL (7.94 × 10^−4^ mg mL^−1^) as previously reported.^75^ This indicates that the PMCL micelles are more thermodynamically stable than PBCL. This implies that PMCL micelles can withstand dilution, minimizing the chances of dissociation of the micelles and premature drug release during circulation.

### Size and morphology of empty and loaded micelles

**Fig. 7:**
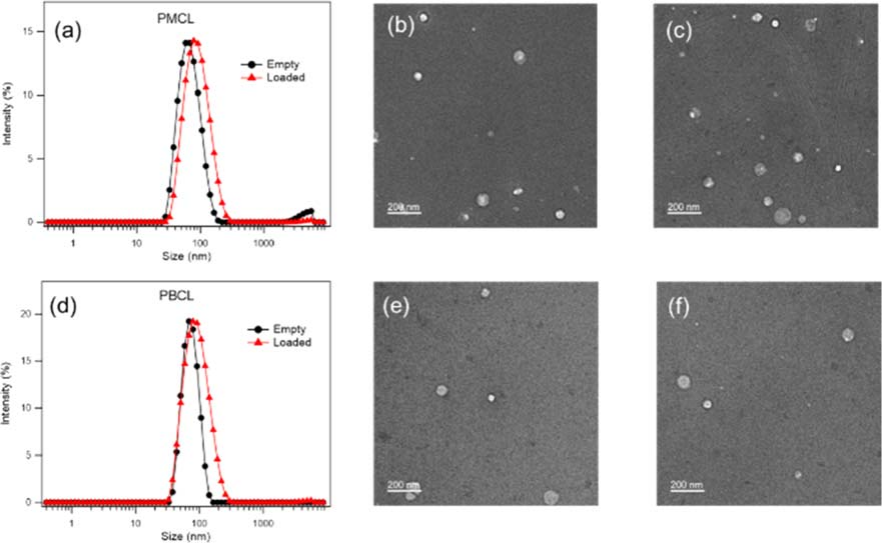
Size (from DLS) and morphology analysis (from TEM showing spherical micelles) of PMCL and PBCL micelles. (a) Average size of empty and DOX-loaded PMCL micelles was 68 nm and 80 nm, (b) TEM of empty PMCL micelles, (c) TEM of DOX-loaded PMCL micelles, (d) average size of empty and DOX-loaded PBCL micelles, were 70 nm and 90 nm, (e) TEM of empty PBCL micelles, (f) TEM of DOX-loaded PBCL micelles. Scale bar: 200 nm.

Both empty and DOX-loaded micelles were prepared using the solvent evaporation method. The micelles were subjected to dynamic light scattering (DLS) to evaluate the hydrodynamic diameters of the micelles and compared to PBCL micelles previously reported from our group.^75^ The PBCL polymer used in this study has a lesser number of repeating units (∼40 DP_n_) in comparison to the previously reported polymer (∼50 DP_n_). The DLS measurement of both empty and DOX-loaded micelles from PMCL and PBCL had a monomodal size distribution as shown in Fig. 7a and d. The size of the empty and DOX-loaded micelles from PMCL were 68 nm and 80 nm, while PBCL had 70 and 90 nm for the empty and DOX-loaded micelles, respectively. The size obtained for the PBCL micelles was consistent with the previously reported value.^75^ The DOX-loaded micelles showed a higher size than the empty micelles from both PMCL and PBCL micelles. The increase in micelles size was a result of the encapsulation of DOX into the core of the micelle.^4,97^ The sizes of the empty micelles from both polymers were similar. However, the DOX-loaded micelles from PMCL micelles were smaller than PBCL micelles. It is reported that the presence of aromatic hydrophobic pendants at the core of micelles tends to engage in π–π stacking with the aromatic system of DOX molecules, leading to close packing of DOX in the micelles.^4,54^ Therefore, micelles core that engages in higher overall interaction will potentially have closer DOX packing, hence smaller micelle size relative to micelles with lower core interaction. Since the maleimide group in the PMCL micelles offers both π–π interactions and hydrogen bonding, a higher overall core interaction with the loaded DOX is expected, leading to a relatively smaller DOX-loaded PMCL micelle size than from the PBCL, which only offers π–π stacking. Such a difference was observed in Fig. 7. Transmission Electron Microscopy (TEM) was used to confirm the morphology of both empty and DOX-loaded micelles (Fig. 7b, c, e, and f). The TEM analysis indicates that all the micelles are spherical, and their sizes were similar to the size from the DLS analysis. An important factor to consider is the stability of micelles during storage in aqueous media; therefore, time-dependent stability (changes in micelle size, aggregation of micelles, and potential formation of smaller micelles) of the empty and loaded micelles from both PMCL and PBCL was analyzed over seven consecutive days (Fig. S17†). From the DLS analysis, no significant change in size or considerable aggregation of micelles was observed. However, there was a slight deviation in the size distribution of empty PMCL micelles on the sixth day, whereas both empty and loaded PBCL micelles remained stable.

### DOX loading and *in vitro* DOX release

**Fig. 8:**
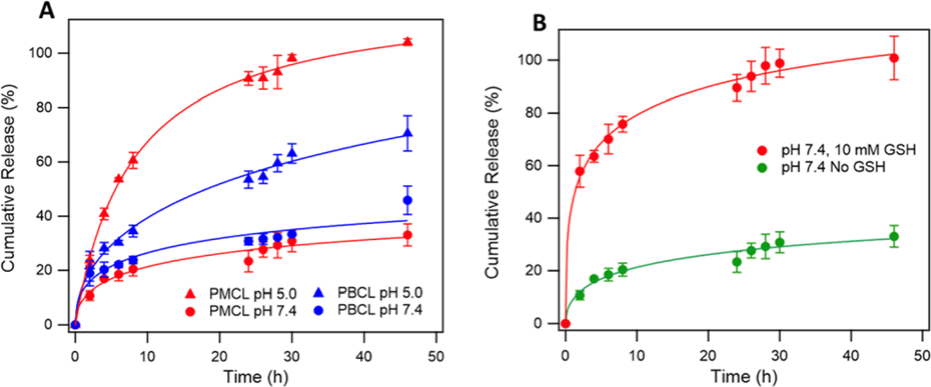
*In vitro* DOX release profile of DOX-loaded PMCL and PBCL micelles (A) micelle exhibited pH-dependent DOX release and higher DOX was released at low pH than at neutral pH. *In vitro* DOX release profile of DOX-loaded PMCL in the presence of 10 mM GSH (B). PMCL micelles exhibited higher DOX release in the presence of GSH.

Since solid tumors have a lower pH than normal cells, polymeric micelles specifically responsive to this low pH to trigger the release of loaded drugs are desirable for controlled drug release applications in biological systems to mitigate the side effects of anticancer drugs.^40^ Interestingly, polymeric micelles from aliphatic polyester such as PCL are reported to be pH-responsive and can be tailored to achieve pH-dependent drug release.^61,98,99^ To analyze the possibility of using PMCL as a drug carrier capable of accumulating at the tumor site through an EPR effect due to the smaller micelles size and releasing a larger amount of the loaded drug as a result of the low pH, DOX was loaded since this anticancer drug is FDA approved for a wide spectrum of tumors.^100^ DOX was encapsulated into the hydrophobic core of the micelles using the solvent evaporation method and dialyzed as described above. The drug loading capacity (DLC) of the PMCL micelles was 3.5%, while PBCL had 2.5%. The DLE was 35.4% and 25% in PMCL and PBCL micelles, respectively (Table 1). The enhancement in DLC of the PMCL can be attributed to the higher overall interaction between the DOX and the 2,3-diiodomaleimide group contributed by both π–π stacking and hydrogen-bonding (from carbonyls and nitrogen) compared with only π–π stacking by the benzyl group of PBCL.^101,102^ The reactivity of amines towards halomaleimide is reported.^103–105^ Such reaction is slower than thiol and leads to a drastic red-shift in the wavelength at which the product absorbs UV-vis light. Since DOX possess an amine group and could potentially react with the 2,3-diiodomaleimide groups, the UV-vis analysis of the DOX-loaded micelle did not show any absorption peak shift as evident in Fig. S19.† This indicates that the DOX did not react with the 2,3-diiodomaleimide groups. However, it is worth noting that stronger interaction between the drug and the polymer could slow the release of the drug molecule,^106^ hence requiring multiple stimuli, either internal or external, to accelerate drug release.^3^ Therefore, slow DOX release would be expected from the DOX-loading PMCL micelles. However, this was not the case, as depicted in Fig. 8 where there was more DOX release from PMBL at a lower pH than PBCL at the same pH. This observation could be a result of the low pH disrupting the H-bonding network between the maleimide group and the DOX thereby weakening the overall interaction hence, facilitating the release of DOX at the low pH.

**Table 1:** DLC and DLE of PMCL and PBCL micelle

DOX-loaded micelles	DLC (%)	DLE (%)
PMCL	3.5 ± 0.34	35.4
PBCL	2.5 ± 0.24	25

The overall higher DOX release at the lower pH than at physiological pH is also attributed to the hydrolyzable ester backbone of PCL, which is well documented.^8,40^ Besides, our group has previously studied and reported the degradation of polycaprolactones at various pH conditions where more was evident at lower pH and after 48 h.^6,107^ According to Fig. 8, PMCL micelles at low pH (5.0) showed almost 100% DOX release after 48 h, while the PBCL micelles had about 60% at the same time. At a pH of 7.4, typical for physiological conditions, both micelles had relatively lower DOX released, whereas the PMCL micelle released about 33% DOX at 48 h while the PBCL released 46%. This low DOX release at the pH of 7.4 from PMCL micelles is more desirable than PBCL micelles for cancer chemotherapy since the lower release at this pH implies lower DOX-mediated toxicity toward healthy cells. This further emphasizes that the property of ε-CL-based polymers can be fine-tuned through the substitution with different functional groups. Moreover, it is worth noting that the release profile of PBCL micelles was consistent with the release profile we reported.^75^

Considering that the maleimide functional group in the PMCL micelles reacted with GSH, it necessitated the need to evaluate the influence of GSH on the release of DOX. This is particularly interesting since the concentration of GSH in cancer cells is considerably higher than normal healthy cells.^21^ To that effect, we analyzed the release profile of PMCL micelles in the presence of 10 mM GSH. As presented in Fig. 8b, the presence of GSH drastically increases the release of DOX from the micelles even at a pH of 7.4 relative to the amount of DOX released without GSH at pH 7.4. The enhanced release might have occurred due to the sudden shift in the ratio between the hydrophobic and hydrophilic blocks of the polymer following the reaction between the thiol of GSH and the maleimide of the PMCL polymer. This confirms that the release of DOX from the PMCL micelles is not only pH-dependent but also GSH-dependent. Therefore, as the DOX-loaded PMCL micelles enter the cancer cells, the lower pH and the higher concentration of GSH will cause a burst release of the loaded drug into the cancer cell.

### *In vitro* cellular uptake and viability

**Fig. 9:**
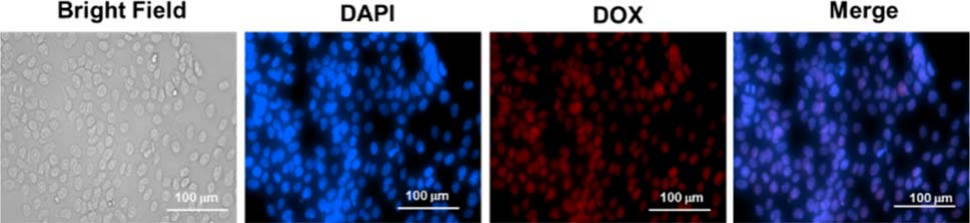
Cellular uptake of PMCL micelles into MDA-MB-231 cells at 37 °C. Fluorescent images were recorded after 4 h of incubation. Images from left to right show a bright field, cells with nuclei staining using DAPI, DOX imaged with RFP filter, and overlays of DAPI and DOX images. Scale bar: 100 μm.

To achieve desirable toxicity, the drug-loaded micelles must enter the cell to release the drugs. Therefore, the cellular uptake of the DOX-loaded micelles into cancer cells was determined. The cellular uptake of DOX-loaded micelles was determined in MDA-MB-231 breast cell lines at 37 °C. The cells were exposed to DOX-loaded PMCL micelles and incubated for 4 h. Following cell fixation, the cell nuclei were stained with DAPI and observed under a Cytation 3 fluorescence microscope (Fig. 9). The merged image, which combines the blue fluorescence (DAPI) representing the stained nuclei and the red fluorescence indicating DOX in the micelles, clearly demonstrates that the DOX-loaded PMCL micelles were internalized into the cancer cells, with DOX predominantly accumulating in the nuclei.

**Fig. 10:**
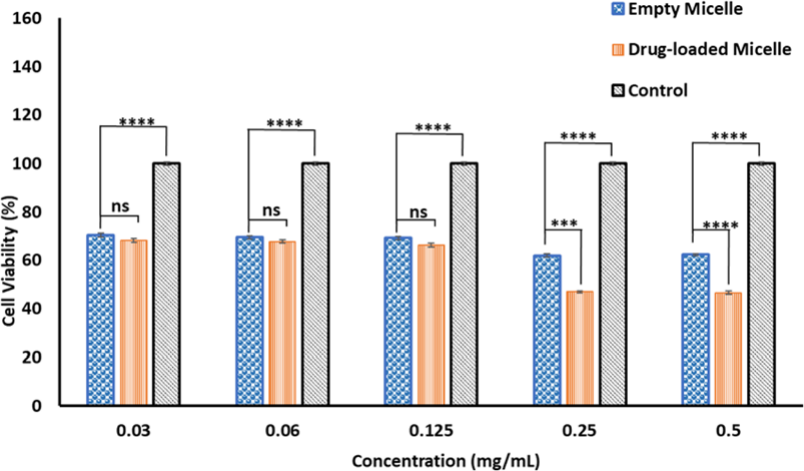
The cytotoxicity of empty and DOX-loaded PMCL micelles at 37 °C. All incubations were done for 24 h, and the cell viability was assessed using MTT assay. A *p*-value < 0.05 was considered statistically significant.

The cytotoxicity of empty and DOX-loaded micelles from PMCL was determined using MTT assay against MDA-MB-231 breast cancer cell lines. The cytotoxicity was determined by varying the dosage of the micelles from 0.5 mg mL^−1^ to 0.03 mg mL^−1^. Cell death was observed at all the tested concentrations for both empty and DOX-loaded micelles (Fig. 10). The DOX-loaded micelles expectedly exhibited the highest cytotoxicity against the cancer cell line, resulting in a cell viability of 45% when the cell was treated with 0.5 mg mL^−1^ concentration. Even at the lowest tested concentration, the cell viability was approximately 65%. Since the PMCL polymer is shown to have a GSH quenching effect, it is expected that the empty micelles will show some cytotoxicity toward the cancer cell lines. This was confirmed by MTT Assay by treating the cells with empty micelles. Cell viability decreased across all the dosages in a concentration-dependent cancer cell death. Even though the PBCL empty micelles had no significant toxicity toward cancer cells,^75^ the toxicity of PMCL empty micelles toward cancer cells is desirable since there are dual paths to the anticancer activity; one from the maleimide group quenching the GSH and weakening the antioxidant defense system of the cancer cells, and from the loaded anticancer drug.

### Intracellular GSH level

**Fig. 11:**
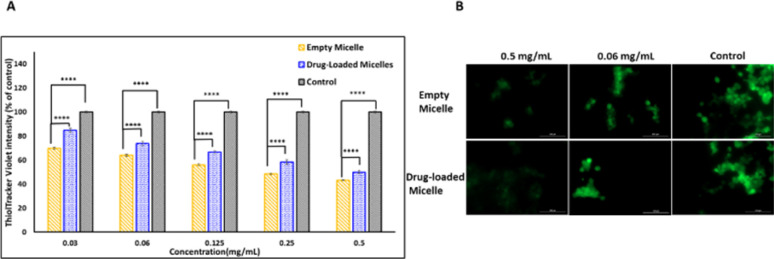
Intracellular GSH-quenching effect using ThiolTracker Violet probe. (A) Intracellular GSH levels after treating MDA-MB-231 with different concentrations of empty and DOX-loaded micelles. (b) Confocal microscope images showing the GSH levels after treatment. Scale bar: 100 μm. A *p*-value < 0.05 was considered statistically significant.

Even though the empty PMCL micelles are proven to quench GSH in a non-cellular environment (Fig. 5), the translation of the same effect must be confirmed in cancer cells. To demonstrate this, the DOX-loaded and empty PMCL micelle's ability to deplete intracellular GSH in MDA-MB-231 breast cell lines was tested. The cell lines were incubated in a 96-well plate for 24 h where each well contained 1 × 10^4^ cells. The Thioltracker Violet assay was employed, where the probe stains the intracellular GSH and produces fluorescence with the intensity dependent on the amount of the GSH present. In this assay, cells containing a large amount of GSH produce fluorescence with higher intensity and *vice versa*. Therefore, cell lines treated with a higher concentration of PMCL polymer – empty or DOX-loaded micelles – are expected to deplete more intracellular GSH, leaving a relatively low concentration of free GSH to be stained by the Thioltracker Violet, hence producing low fluorescence intensity. This trend was evident in both empty and DOX-loaded micelles, as illustrated in Fig. 11a where the 0.5 mg mL^−1^ polymer had the highest GSH quenching effect than with 0.03 mg mL^−1^ concentration. However, the GSH quenching effect was found to be higher in the empty micelles than in the DOX-loaded micelles at all the tested concentrations. For instance, 0.5 mg mL^−1^ polymer concentration exhibited about 43% reduction in intracellular GSH by the empty micelle, while the DOX-loaded micelles had about 50%. The observation can be attributed to the maleimide pendants being less restricted by the presence of cargo molecules in the core of the micelle, unlike the DOX-loaded micelles. The presence of DOX in the DOX-loaded micelles reduces the frequency of effective collision between the intracellular GSH and the maleimide pendants, resulting in a slower reaction and lowering the GSH-quenching effect at a given time. Nevertheless, the ability of the DOX-loaded micelles to quench intracellular GSH was still significant even at the lowest polymer concentration, where about 16% GSH was quenched.

The intracellular GSH levels in the cancer cells were also visualized using Thioltracker Violet as presented in Fig. 11b. In the Figure, cells without any micelles (control) showed a stronger fluorescence intensity, indicating a higher level of GSH in the cancer cell lines. However, the fluorescence intensity dimmed when the cell lines were treated with micelles, demonstrating a decline in the intracellular GSH level due to the quenching effect of the PMCL polymer from both empty and DOX-loaded micelles. It is imperative to note that the 2,3-diiodomaleimide groups can react with other intracellular thiols such as thiol-containing peptides. However, since the 2,3-diiodomaleimide groups are buried inside the core of the micelles they are protected from the intracellular environment therefore limiting the propensity at which other thiols can react with the 2,3-diiodomaleimide groups. Moreover, thiols macromolecules with large molecular weight due to their size cannot readily diffuse into the micelles to respond with the 2,3-diiodomaleimide groups.

## Conclusions

In conclusion, we synthesized a novel 2,3-diiodomaleimide functionalized amphiphilic polycaprolactone block copolymer PMCL and examined its ability to quench glutathione as well as studied its effectiveness in loading and releasing the anticancer drug, doxorubicin. Only a handful of reports have demonstrated the use of maleimides to deplete glutathione. Even so, unsubstituted maleimide capable of reacting with only a single GSH at a time is the most common. This is the first time a biodegradable polymer functionalized with maleimides capable of quenching more than one molecule of GSH has been reported. Our synthetic approach for maleimide-functionalized PCL is novel, as maleimide groups were never previously attached to caprolactone monomers before this work. The synthesized PMCL polymer reduced glutathione levels *in vitro*, leading to significant cancer cell death. Additionally, since the PBCL is an amphiphilic polymer capable of forming micelles, DOX was loaded at exceptionally higher DLC and DLE than the reference polymer, PBCL. So far, PMCL has the highest DLC among the γ-substituted polycaprolactones we have reported. This significant DLC has promising applications in drug delivery, extending beyond its initial intention of quenching glutathione in cancer cells. The release profile of the PMCL polymer is also remarkable, enabling drug release in a pH-dependent manner and the presence of glutathione. This dual-stimuli responsive behavior is significant in the context of cancer cells, which are known for elevated glutathione levels and lower pH. Future studies on maleimide-based drug delivery systems will focus on improving the targeting capabilities to minimize potential toxicity to healthy cells.

## Data availability

Compound characterization data and experimental procedures are available in the ESI.†

## Author contributions

Godwin K. Babanyinah and Abhi Bhadran contributed to the conception, experimental design, data acquisition, and manuscript preparation. Himanshu Polara contributed to data acquisition, data analysis, and manuscript preparation. Hanghang Wang and Tejas Shah contributed to the data analysis and editing manuscript. Michael C. Biewer and Mihaela C. Stefan contributed to the conceptualization, manuscript drafting, and editing.

## Conflicts of interest

There are no conflicts to declare.

## Supplementary Material

SC-015-D4SC01625D-s001
