# Supplementary material for: Maleimide functionalized polycaprolactone micelles for glutathione quenching and doxorubicin delivery
Source: Chem Sci. 2024 May 24;15(26):9987–10001. doi: 10.1039/d4sc01625d (PMC11220601; doi:10.1039/d4sc01625d)
Supplement: SC-015-D4SC01625D-s001 [file SC-015-D4SC01625D-s001.pdf]

Electronic Supplementary Material (ESI) for Chemical Science.
This journal is © The Royal Society of Chemistry 2024

Maleimide Functionalized Polycaprolactone Micelles for Glutathione Quenching and Doxorubicin Delivery

Godwin K. Babanyinah,[a] Abhi Bhadran,[a] Himanshu Polara,[b] Hanghang Wang,[b] Tejas Shah,[b] Michael C. Biewer,* and Mihaela C. Stefan*

Department of Chemistry and Biochemistry, University of Texas at Dallas, Richardson, TX, USA

[a] These authors contributed equally to the work.

* Correspondence to Mihaela C Stefan at mihaela@utdallas.edu

Supporting Information

Synthesis of Monomer

Synthesis of tert-butyl (3-((4-oxocyclohexyl)oxy)propyl)carbamate. To a solution of 2.0 g (86 mmol) NaH in 25 mL N, N-dimethylformamide (DMF), 1,4-cyclohexanediol (10.0 g, 86 mmol) dissolved in 15 mL DMF was added and stirred in N_2 environment at room temperature. After stirring for 3 hours, a solution of *tert*-butyl (3-bromopropyl)carbamate (20.5 g, 86 mmol) dissolved in 15 mL DMF was added dropwise in ice cold condition. The solution was then brought back to room temperature and stirred under N_2 for 12 h. Once the reaction was completed, the solution was quenched using 2 N HCl (100 mL) followed by extraction with DCM (3 x 100 mL). The DCM layers were combined, dried with anhydrous magnesium sulfate, concentrated with vacuo, and redissolved in 15 mL DCM. The crude product was added dropwise to a solution of pyridinium chlorochromate (5.4 g, 25 mmol) in DCM at 0 ˚C while stirring under N_2 atmosphere. The solution was brought to room temperature and stirred for 12 h. After the complete consumption of the starting material confirmed by TLC, the reaction mixture was diluted with 100 mL of DCM, followed by an aqueous wash (3 x 100 mL). The organic layers were dried with anhydrous magnesium sulfate and concentrated with vacuo. The crude mixture was purified through column chromatography with ethyl acetate to hexane of 1:1. to produce yellow oil (yield = 90 %). ^{1}H NMR (500 MHz, $CDCl_3$) δ 4.91 (s, 1H), 3.68 (tt, *J* = 5.6, 2.7 Hz, 1H), 3.57 (t, *J* = 5.8 Hz, 2H), 3.25 (q, *J* = 6.3 Hz, 2H), 2.56 (ddd, *J* = 15.6, 10.7, 5.6 Hz, 2H), 2.25 (dt, *J* = 14.7, 5.4 Hz, 2H), 2.08 (dq, *J* = 11.4, 5.4 Hz, 2H), 1.96 – 1.86 (m, 2H), 1.79 (p, *J* = 6.1 Hz, 2H), 1.42 (s, 9H). ^{13}C NMR (500 MHz, CDCl3) δ 211.25, 156.11, 79.20, 73.11, 67.00, 39.01, 37.21, 30.58, 30.12, 28.53.

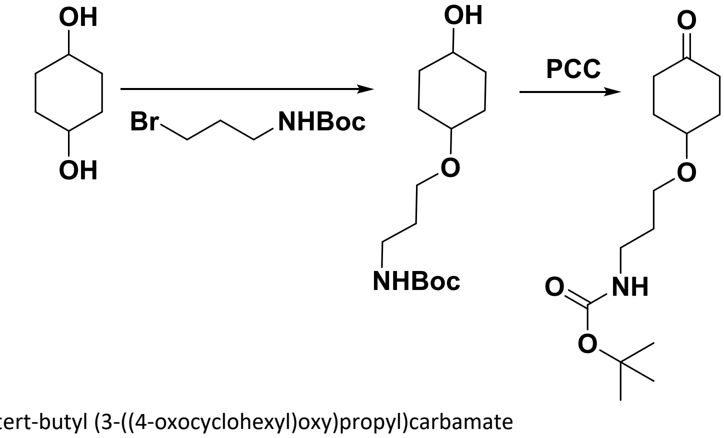

Scheme S1. Synthesis of tert-butyl (3-((4-oxocyclohexyl)oxy)propyl)carbamate

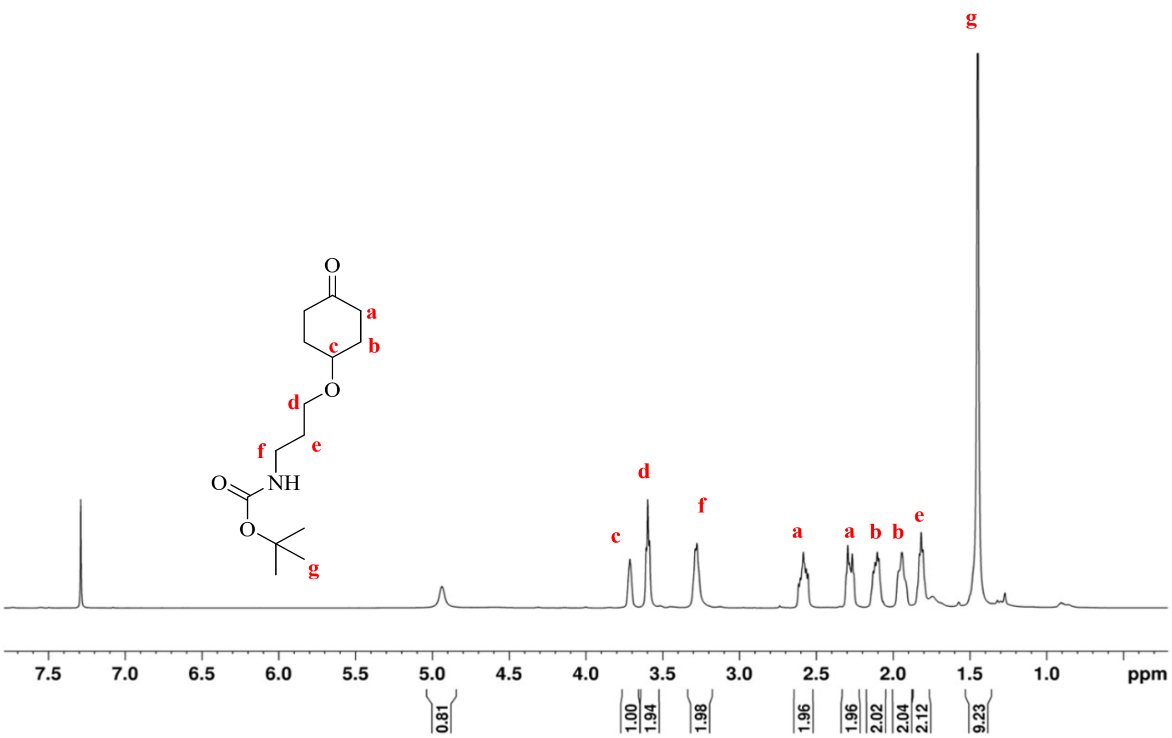

Figure S1. ^{1}H NMR spectrum of *tert*-butyl (3-((4-oxocyclohexyl)oxy)propyl)carbamate.

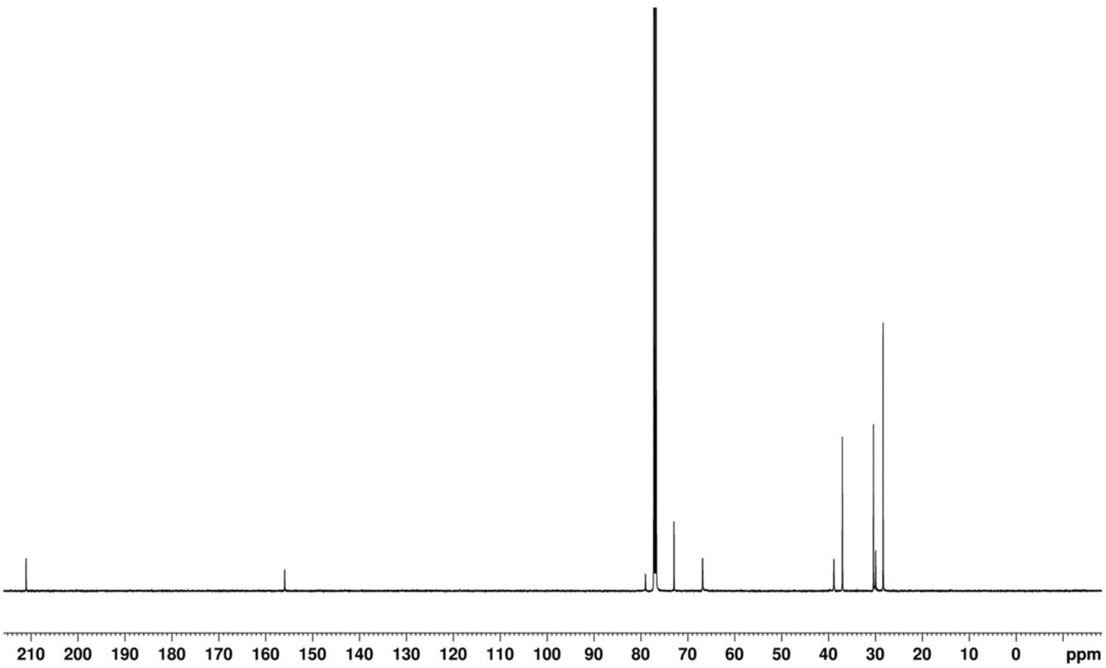

Figure S2. ^{13}C NMR spectrum of *tert*-butyl (3-((4-oxocyclohexyl)oxy)propyl)carbamate.

Synthesis of 4-(3-aminopropoxy)cyclohexan-1-one. Tetrafluoro acetic acid (7 mL) was added to a solution of *tert*-butyl (3-((4-oxocyclohexyl)oxy)propyl)carbamate (4.9 g, 18 mmol) dissolved in 20 mL DCM and stirred at room temperature for 5 h. The solution was then concentrated via vacuo producing a yellow oil (yield = 100 %). ^{1}H NMR (500 MHz, $CDCl_3$) δ 7.96 (s, 2H), 3.74 (s, 1H), 3.67 (s, 2H), 3.15 (s, 2H), 2.49 (d, *J* = 13.6 Hz, 2H), 2.26 (d, *J* = 13.8 Hz, 2H), 2.03 (d, *J* = 14.2 Hz, 6H). ^{13}C NMR (500 MHz, $CDCl_3$) δ 211.28, 73.95, 66.82, 39.14, 37.09, 30.25, 27.41.

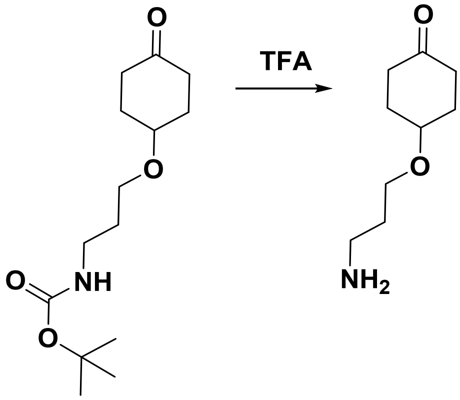

Scheme S2. Synthesis of 4-(3-aminopropoxy)cyclohexan-1-one.

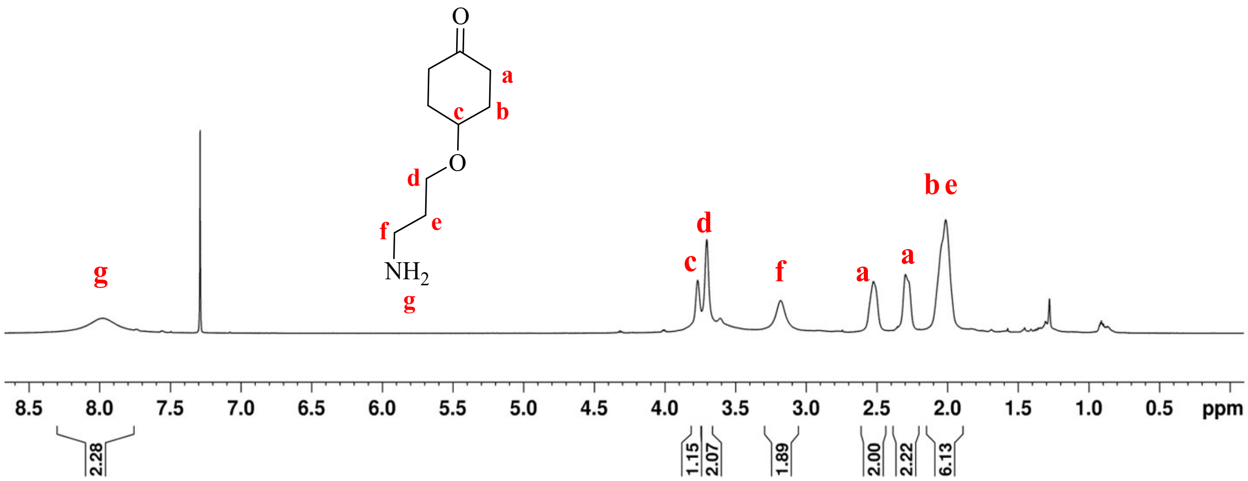

Figure S3. ^{1}H NMR spectrum of 4-(3-aminopropoxy)cyclohexan-1-one.

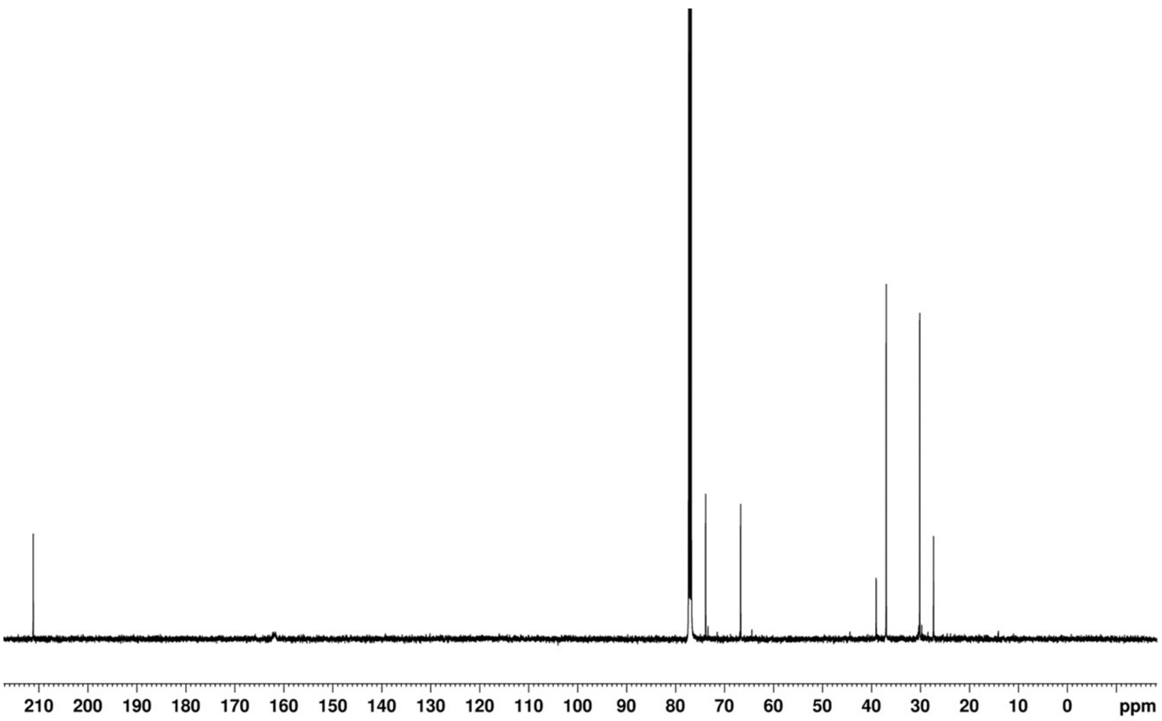

Figure S4. ^{13}C NMR spectrum of 4-(3-aminopropoxy)cyclohexan-1-one.

Synthesis of 3,4-dichloro-1-(3-((4-oxocyclohexyl)oxy)propyl)-1H-pyrrole-2,5-dione. To a solution of 4-(3-aminopropoxy)cyclohexan-1-one (3.1 g, 18 mmol) in 30 mL glacial acetic acid, 3,4-dichlorofuran-2,5-dione (3.0 g, 18 mmol) was added in portions and reflexed for 12 hours. The reaction mixture was concentrated under vacuo and the product was purified through column chromatography with ethyl acetate: hexane (3:7) as eluent. The product was then concentrated under vacuo producing yellow viscous oil (yield 57 %). ^{1}H NMR (500 MHz, $CDCl_3$) δ 3.76 (t, *J* = 6.5 Hz, 2H), 3.63 (tt, *J* = 5.9, 3.1 Hz, 1H), 3.51 (t, *J* = 5.7 Hz, 2H), 2.51 (ddd, *J* = 15.4, 10.1, 5.8 Hz, 2H), 2.24 (dt, *J* = 14.4, 5.8 Hz, 2H), 1.98 (td, *J* = 13.9, 7.5 Hz, 2H), 1.96 – 1.85 (m, 4H). ^{13}C NMR (500 MHz, $CDCl_3$) δ 211.22, 163.23, 133.44, 73.44, 66.00, 37.40, 37.25, 30.52, 28.80.

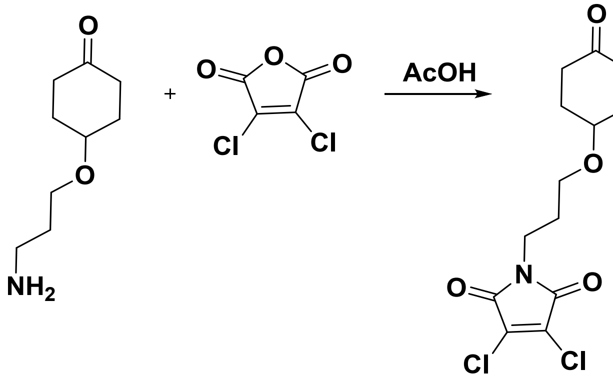

Scheme S3. Synthesis of 3,4-dichloro-1-(3-((4-oxocyclohexyl)oxy)propyl)-1H-pyrrole-2,5-dione

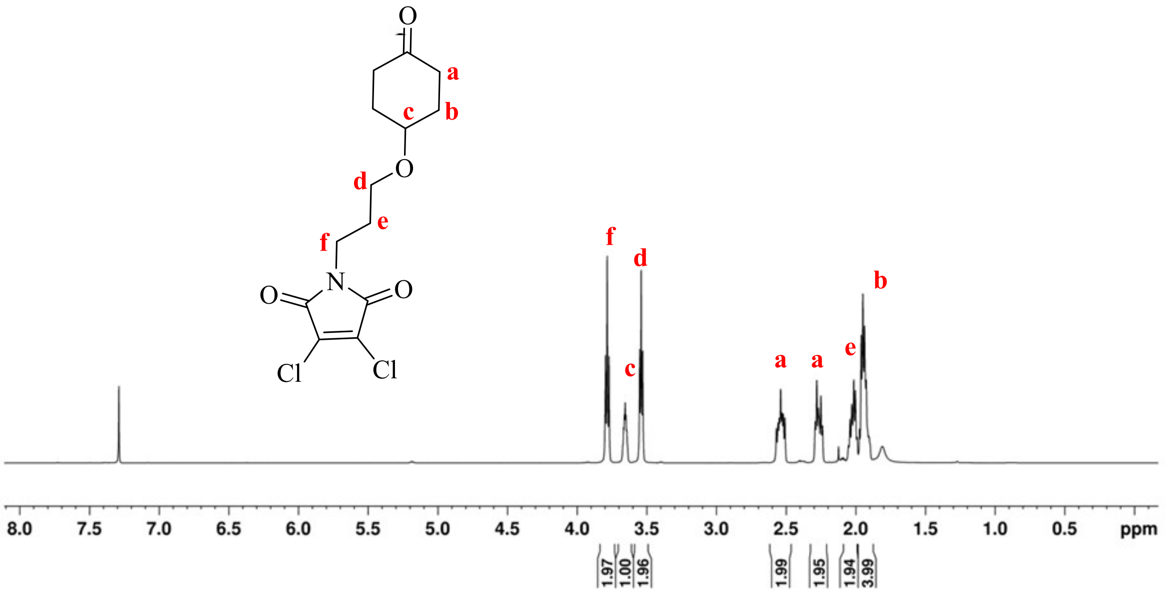

Figure S5. ^{1}H NMR spectrum of 3,4-dichloro-1-(3-((4-oxocyclohexyl)oxy)propyl)-1H-pyrrole-2,5-dione.

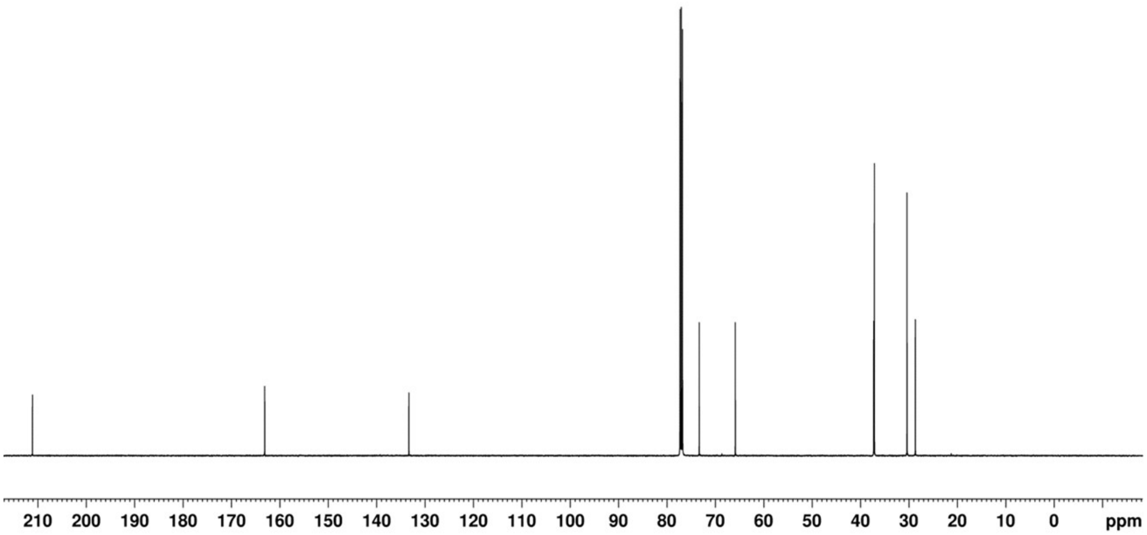

Figure S6. ^{13}C NMR spectrum of 3,4-dichloro-1-(3-((4-oxocyclohexyl)oxy)propyl)-1H-pyrrole-2,5-dione.

Synthesis of 3,4-diiodo-1-(3-((4-oxocyclohexyl)oxy)propyl)-1*H*-pyrrole-2,5-dione. Sodium iodide (NaI, 4.6 g, 30 mmol) was added in portions to a solution of 3,4-dichloro-1-(3-((4-oxocyclohexyl)oxy)propyl)-1H-pyrrole-2,5-dione (3.3 g, 10 mmol) in 20 mL acetonitrile. The solution was refluxed at 90 °C die 12 h. A small amount of the reaction mixture was then analyzed by ^{13}C NMR to confirm the complete consumption of the starting material. Once the reaction was completed, the solvent was evaporated and redissolved in 100 mL DCM. The crude product was washed three times each with 100 mL DI water. The organic layer was combined, dried with anhydrous magnesium sulfate, and concentrated through vacuo to produce yellow oil (yield = 97 %). ^{1}H NMR (500 MHz, $CDCl_3$) δ 3.79 (t, *J* = 6.6 Hz, 2H), 3.62 (tt, *J* = 5.9, 3.1 Hz, 1H), 3.51 (t, *J* = 5.7 Hz, 2H), 2.51 (ddd, *J* = 15.4, 10.3, 5.8 Hz, 2H), 2.23 (dt, *J* = 14.5, 5.8 Hz, 2H), 2.00 (dq, *J* = 11.9, 5.8 Hz, 2H), 1.95 – 1.84 (m, 4H). ^{13}C NMR (500 MHz, $CDCl_3$) δ 211.35, 166.58, 117.54, 73.34, 66.06, 38.22, 37.30, 30.59, 28.90.

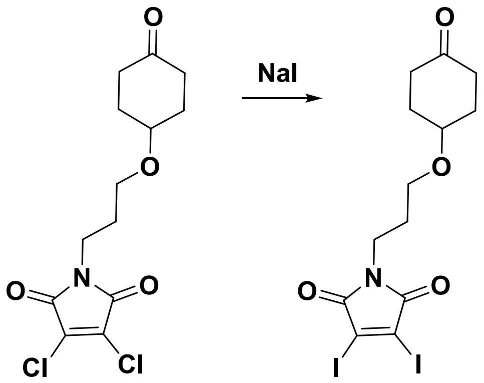

Scheme S4. Synthesis of 3,4-diiodo-1-(3-((4-oxocyclohexyl)oxy)propyl)-1*H*-pyrrole-2,5-dione

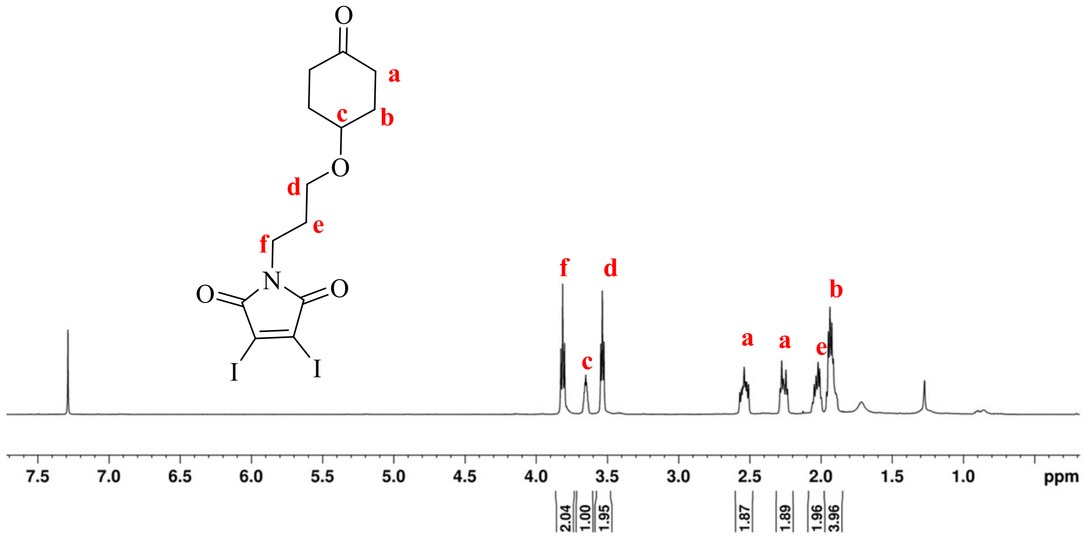

Figure S7. ^{1}H NMR spectrum of 3,4-diiodo-1-(3-((4-oxocyclohexyl)oxy)propyl)-1H-pyrrole-2,5-dione.

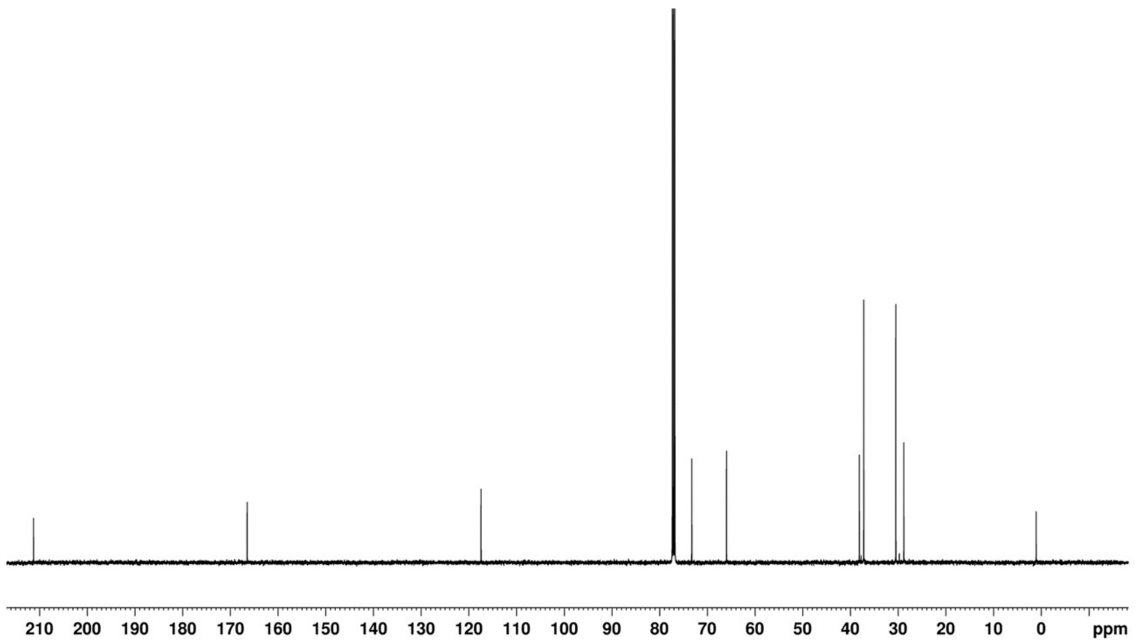

Figure S8. ^{13}C NMR spectrum of 3,4-diiodo-1-(3-((4-oxocyclohexyl)oxy)propyl)-1H-pyrrole-2,5-dione.

Synthesis of 3,4-diiodo-1-(3-((7-oxooxepan-4-yl)oxy)propyl)-1H-pyrrole-2,5-dione (DIMCL). To a solution of 3,4-diiodo-1-(3-((4-oxocyclohexyl)oxy)propyl)-1H-pyrrole-2,5-dione (4.8 g, 10.0 mmol) in 30 mL DCM, m-chloroperoxybenzoic acid (3.5 g, 10.0 mmol) was added in portions under ice cold conditions. The solution was brought to room temperature and kept stirring for 12 h. After a complete reaction, the solution was quenched with potassium carbonate and stirred for another 3 h. About 30 mL of DCM was added and washed three times with 100 mL saturated potassium carbonate DI water. The organic layer was dried with anhydrous magnesium sulfate and concentrated under vacuo. The crude product was purified through column chromatography with ethyl acetate: hexane: 1:3 to obtain a yellowish solid product (yield = 80 %). ^{1}H NMR (500 MHz, $CDCl_3$) δ 4.49 – 4.41 (m, 1H), 4.05 (ddd, *J* = 13.1, 6.2, 1.9 Hz, 1H), 3.77 (t, *J* = 6.5 Hz, 2H), 3.60 (tt, *J* = 5.6, 2.8 Hz, 1H), 3.53 – 3.37 (m, 2H), 2.92 (ddd, *J* = 14.0, 11.9, 1.8 Hz, 1H), 2.40 (ddd, *J* = 14.3, 8.4, 1.6 Hz, 1H), 2.06 – 1.76 (m, 6H). ^{13}C NMR (126 MHz, $CDCl_3$) δ 176.08, 166.57, 117.51, 74.55, 65.80, 65.76, 63.50, 37.96, 33.96, 28.89, 27.97, 27.41. MALDI-TOF MS M.W. : 542.85 g mol^{-1}; calculated M.W. : 518.90 g mol^{-1}; mass account for Na^{+} ion: 22.99.

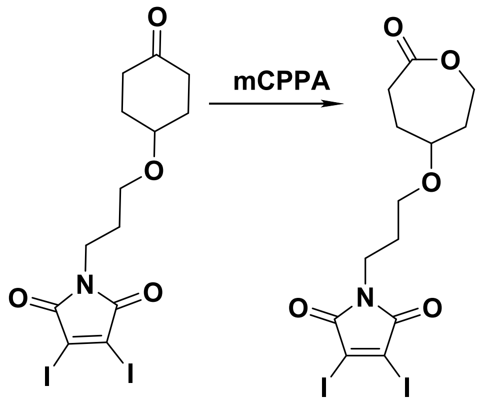

Scheme S5. Synthesis DIMCL.

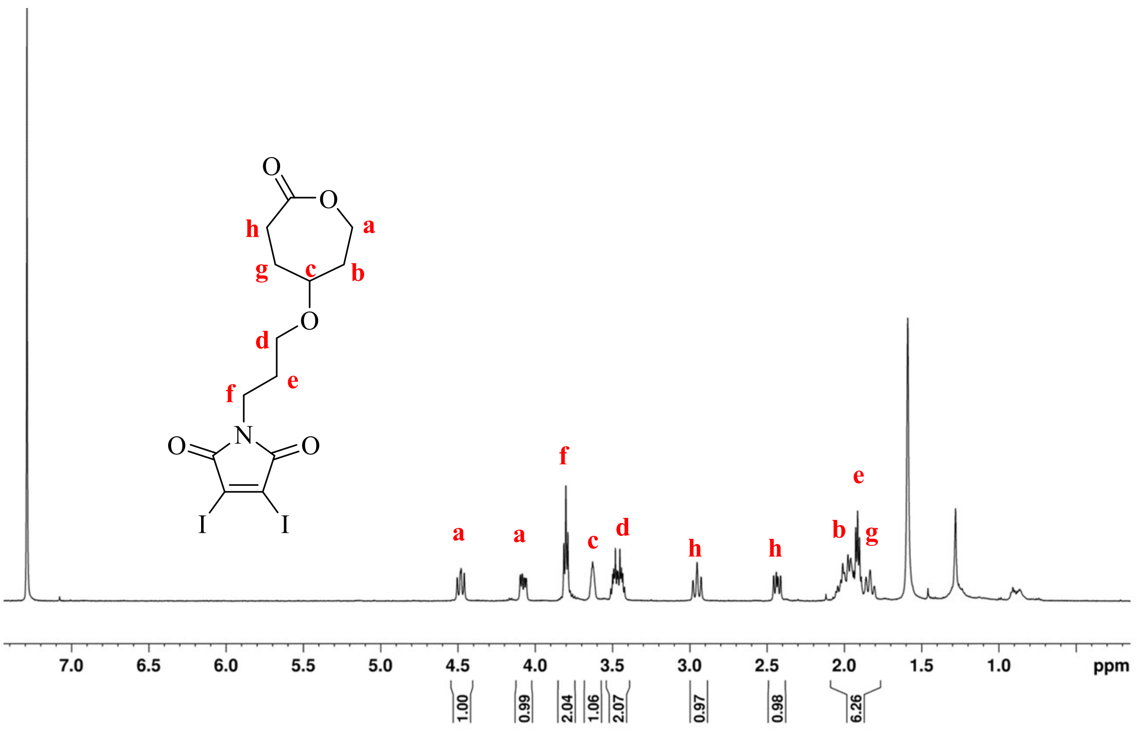

Figure S9. ^{1}H NMR spectrum of DIMCL monomer.

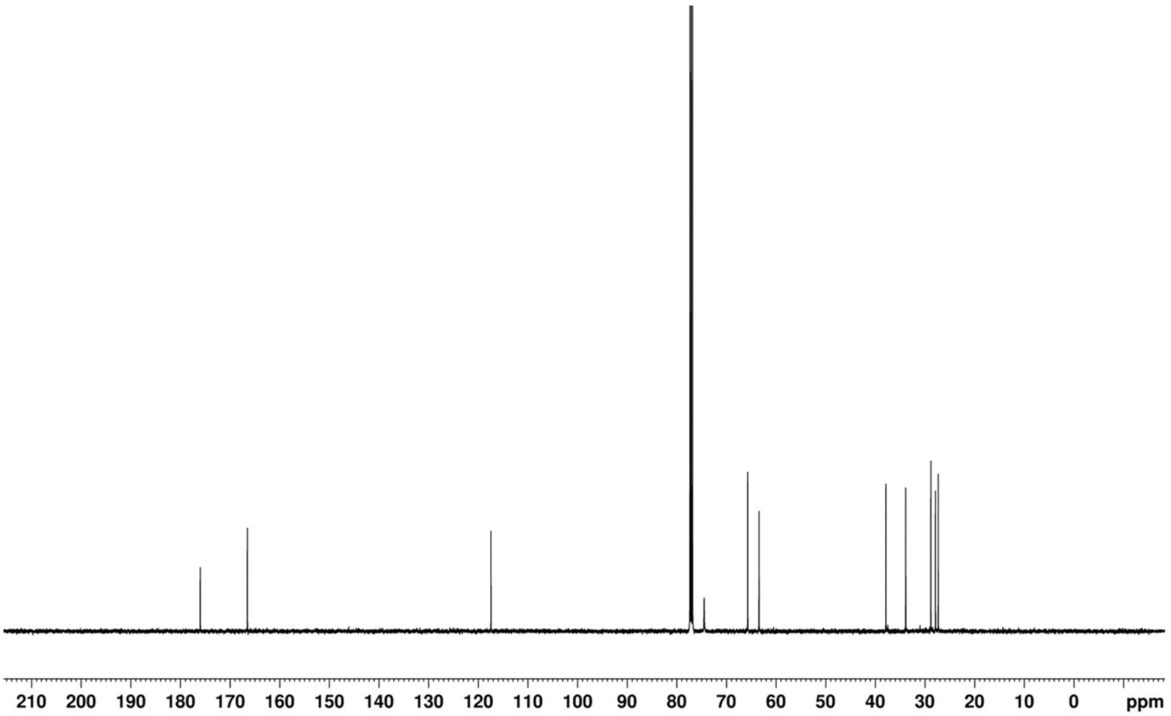

Figure S10. ^{13}C NMR spectrum of DIMCL monomer.

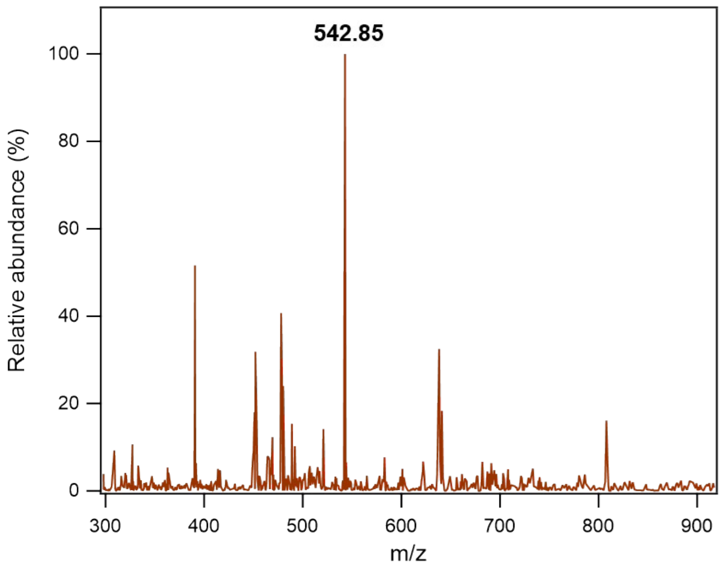

Figure S11. MALDI-TOF mass analysis of DIMCL monomer.

Synthesis of 3,4-bis(benzylthio)-1-(3-((7-oxooxepan-4-yl)oxy)propyl)-1H-pyrrole-2,5-dione. In a round-bottomed flask, 3,4-diiodo-1-(3-((7-oxooxepan-4-yl)oxy)propyl)-1H-pyrrole-2,5-dione (200 mg, 0.38 mmol) was dissolved in 3 mL DCM. Benzylthiol (94.7 mg, 0.77 mmol) was added, and the solution was stirred at room temperature for about 2 minutes. To this solution, triethylamine (50 µL, 0.12 mmol) was added dropwise. The solution was then stirred for 30 minutes. The solvent was removed in vacuo and the product was isolated via column chromatography with ethyl acetate: hexane = 1:4 as a yellow solid (90 %). ^{1}H NMR (500 MHz, $CDCl_3$) δ 7.12-7.34 (m, 10H), (ddd, *J* = 7.8, 1.5, 1.2, 0.5 Hz)), 4.49 – 4.48 (m, 1H), 4.44 (s, 4H) 4.05 (ddd, *J* = 13.1, 6.2, 1.9 Hz, 1H), 3.77 (t, *J* = 6.5 Hz, 2H), 3.60 (tt, *J* = 5.6, 2.8 Hz, 1H), 3.53 – 3.37 (m, 2H), 2.92 (ddd, *J* = 14.0, 11.9, 1.8 Hz, 1H), 2.40 (ddd, *J* = 14.3, 8.4, 1.6 Hz, 1H), 2.06 – 1.76 (m, 6H).

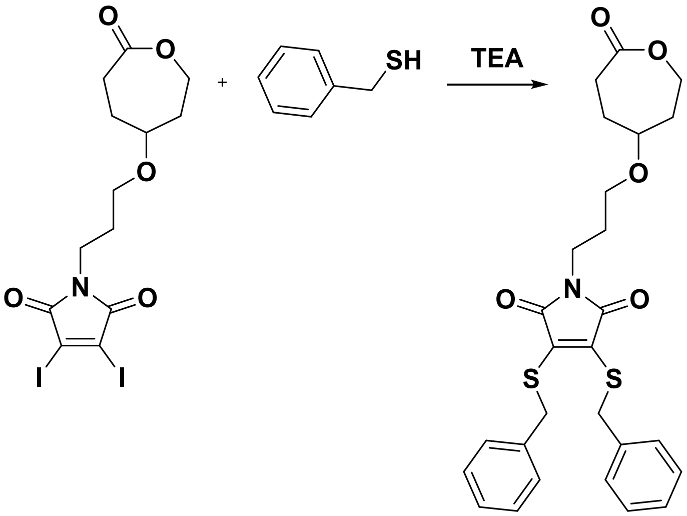

Scheme S6. 3,4-bis(benzylthio)-1-(3-((7-oxooxepan-4-yl)oxy)propyl)-1H-pyrrole-2,5-dione.

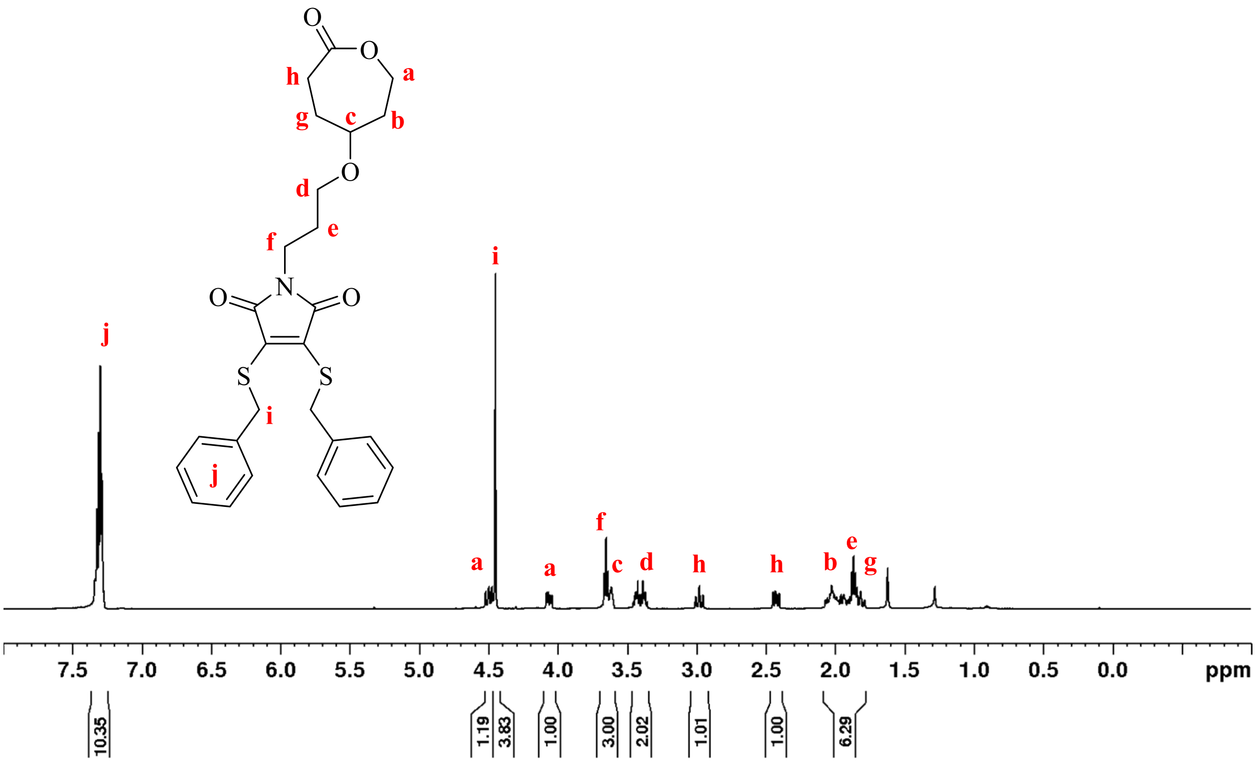

Figure S12. ^{1}H NMR of 3,4-bis(benzylthio)-1-(3-((7-oxooxepan-4-yl)oxy)propyl)-1H-pyrrole-2,5-dione.

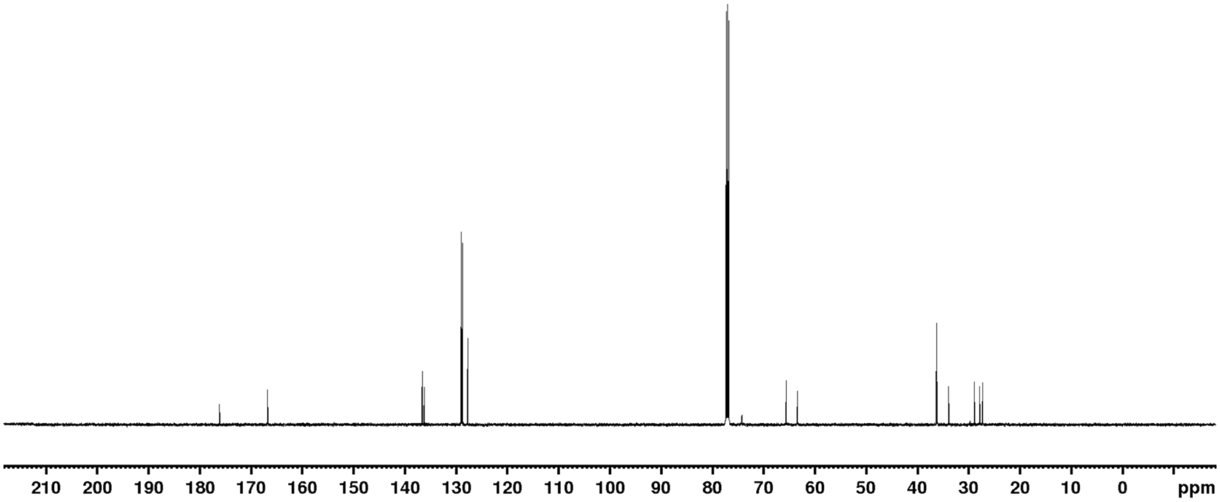

Figure S13. ^{13}C NMR 3,4-bis(benzylthio)-1-(3-((7-oxooxepan-4-yl)oxy)propyl)-1H-pyrrole-2,5-dione.

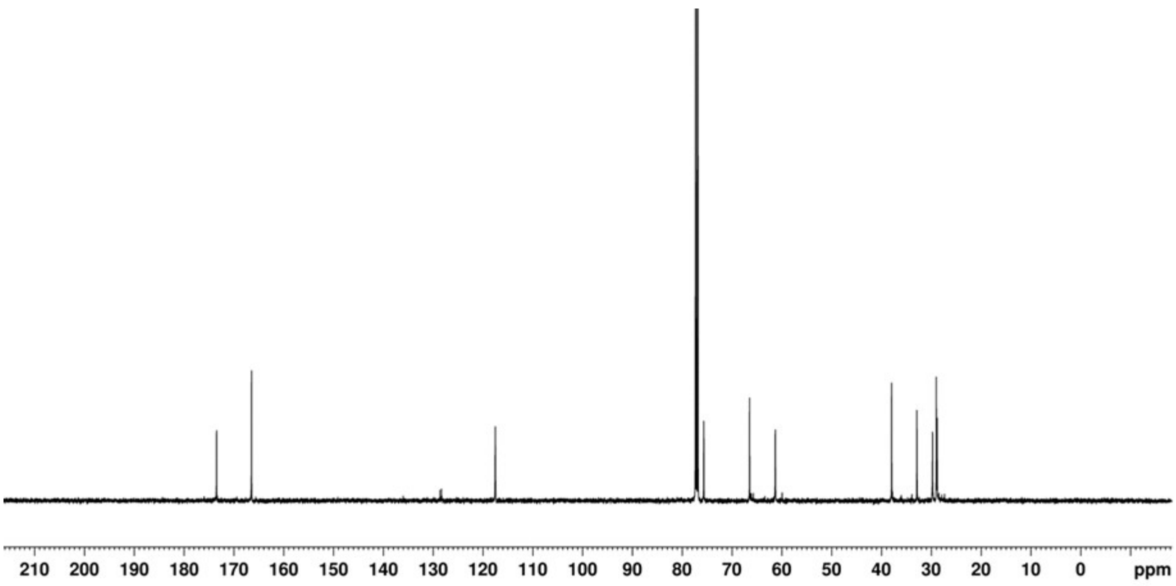

Figure S14. ^{13}C NMR spectrum of homopolymer synthesized from DIMCL monomer.

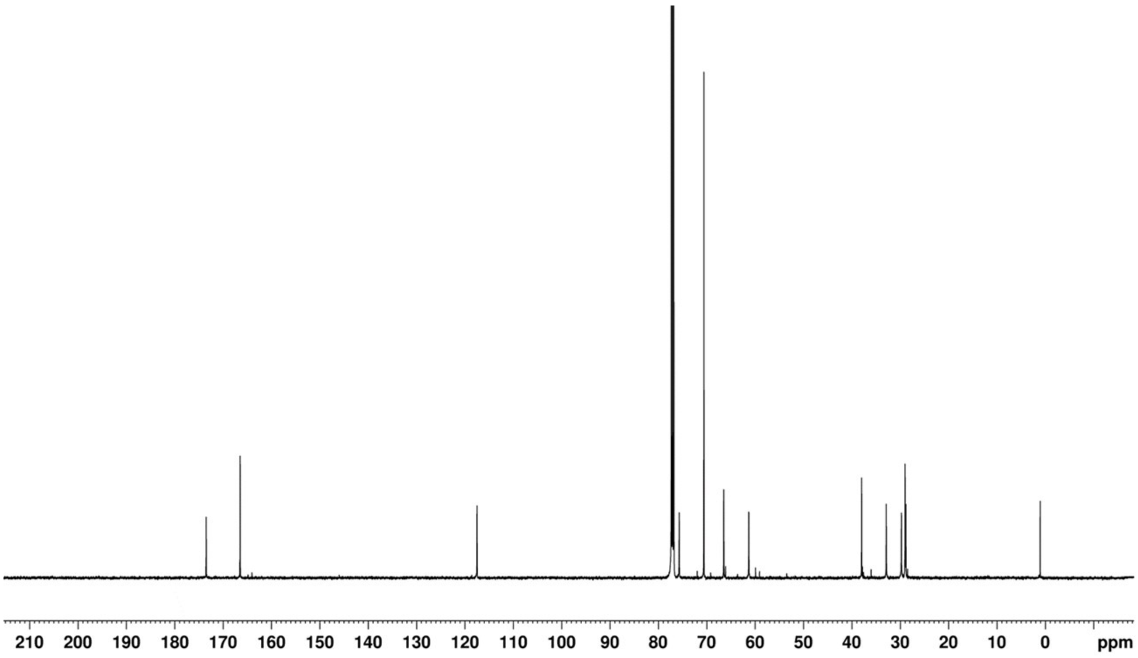

Figure S15. ^{13}C NMR spectrum of amphiphilic block copolymer PMCL.

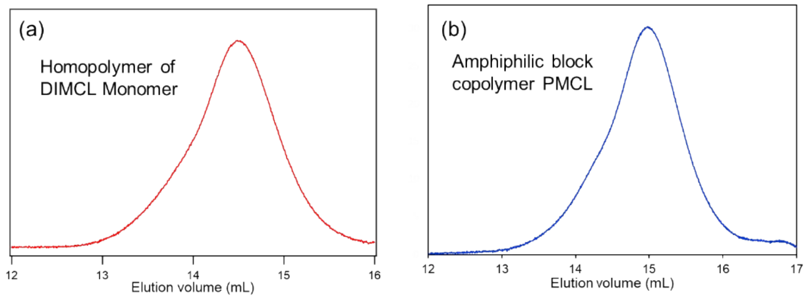

Figure S16. SEC traces of (a) homopolymer made from DIMCL monomer and (b) amphiphilic block copolymer PMCL.

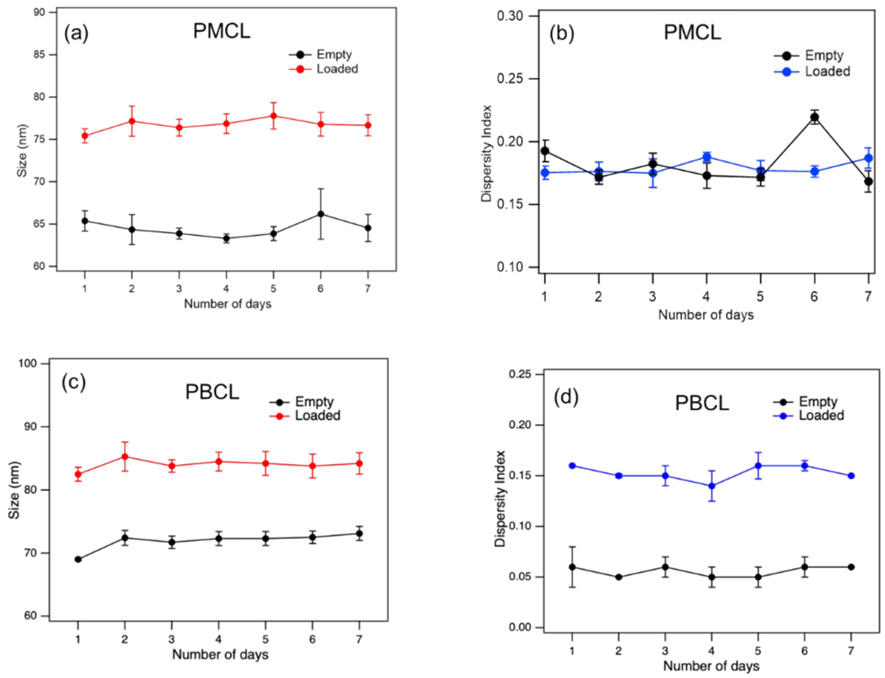

Figure S17. Storage stability of empty and DOX-loaded micelles of PMCL and PBCL analyzed by DLS.

GSH Calibration

The quantification of glutathione was determined using Ellman's test, where the procedure was followed with slight modification from Moser *et al*.[65] In the test, 0.2 mL of freshly prepared GSH standard solution with concentration from 0.1 mM to 1.0 mL was added to 2.5 mL PBS (pH 7.4) and incubated for 30 minutes. Afterward, 0.3 mL of Ellman's reagent (10 mM) was added to each solution and incubated for another 10 minutes. The absorbance at 412 nm was measured and recorded, from which the GSH standard curve was plotted with absorbance against the concentration of the GSH.

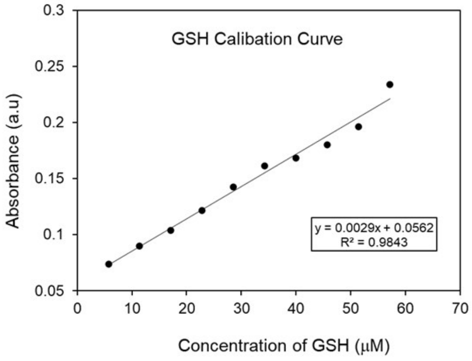

Figure S18. Calibration curve of GSH in PBS. The absorbance was measured at 412 nm after the addition of Ellman's reagent to each concentration.

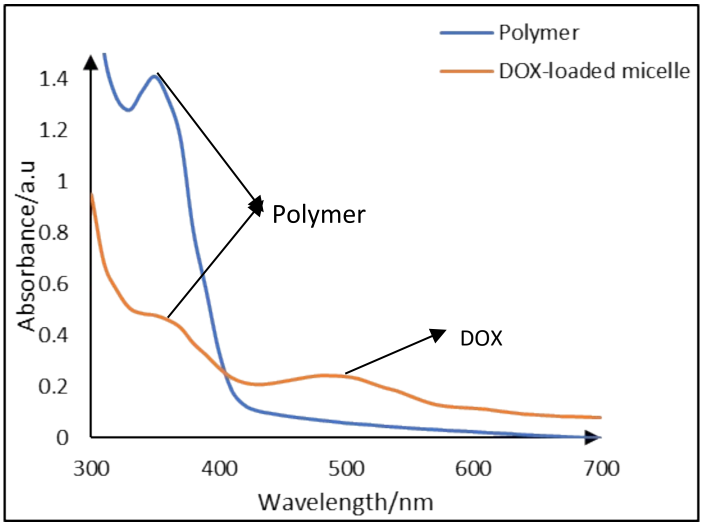

Figure S19. Uv-vis spectrum of PMCL and DOX-loaded micelles. PMCL has aborption peak at 350 nm and the peak at 500 nm IS the absorption peak for DOX. There was no peak shift in the polymer indicating that the amine from the DOX is not reacting with the 2,3-diiodomaleimide groups.
